# A Designed *α*‐GalCer Analog Promotes Considerable Th1 Cytokine Response by Activating the CD1d‐iNKT Axis and CD11b‐Positive Monocytes/Macrophages

**DOI:** 10.1002/advs.202000609

**Published:** 2020-06-08

**Authors:** Juan Ma, Peng He, Chuanfang Zhao, Quanzhong Ren, Zheng Dong, Jiahuang Qiu, Yang Jing, Sijin Liu, Yuguo Du

**Affiliations:** ^1^ State Key Laboratory of Environmental Chemistry and Ecotoxicology Research Center for Eco‐Environmental Sciences Chinese Academy of Sciences Beijing 100085 P. R. China; ^2^ School of Environmental Sciences University of Chinese Academy of Sciences Beijing 100049 P. R. China; ^3^ School of Chemical Sciences University of Chinese Academy of Sciences Beijing 100049 P. R. China; ^4^ National Engineering Research Center for Carbohydrate Synthesis Jiangxi Normal University Nanchang Jiangxi 330022 China

**Keywords:** antitumor activity, CD11b monocytes, CD1d, Th1‐biased immune responses, *α*‐GalCer analogs

## Abstract

Selective helper T cell 1 (Th1) priming agonists are a promising area of investigation for immunotherapeutic treatment of various diseases. *α*‐galactosylceramide (*α*‐GalCer, KRN7000), a well‐studied Th1‐polarizer, simultaneously induces helper T cell 2 (Th2)‐type responses, which is a major drawback for its clinical applications. Based on surflex‐docking computation, *α*‐GalCer‐diol, with added hydroxyl groups in the acyl chain, is designed and synthesized. Structural analyses reveal stronger affinity between *α*‐GalCer‐diol and cluster of differentiation 1d (CD1d), leading to enhanced antigen presentation by dendritic cells (DCs) and self‐activation, as reflected by tight binding of the T‐cell receptor (TCR)/KRN7000/CD1d ternary complex and elevated production of interleukin 12 (IL‐12) and interferon‐*γ* (IFN‐*γ*). Consequently, invariant natural killer T cells (iNKTs) are activated and exhibit an improved Th1‐type cytokine profile ex vivo and in vivo. Different from KRN7000, *α*‐GalCer‐diol markedly boosts the expansion of the CD11b^+^ subpopulation and enhances IFN‐*γ* content in CD11b^+^ cells. These reinforced Th1‐type responses collectively endow *α*‐GalCer‐diol more robust antitumor activity in a xenograft animal model using B16‐F10 melanoma cells. Together, the data demonstrate a new mechanism through which *α*‐GalCer‐diol induces stronger Th1‐type responses by stimulating CD11b^+^ leukocyte expansion and DC‐conducted CD1d‐restricted and TCR‐mediated iNKT activation. Hence, this study may facilitate the development of novel Th1 priming agonists.

## Graphical abstract

Designed *α*‐galactosylceramide (*α*‐GalCer)‐diol generates higher affinity with cluster of differentiation 1d (CD1d) by forming hydrogen bonds with polar residues in A’ pocket of CD1d, leading to stronger helper T cell 1 (Th1)‐type responses and antitumor activity through a novel mechanism of stimulating CD11b^+^ leukocyte expansion and Th1‐priming state. This study may facilitate the development of new Th1 priming agonists.

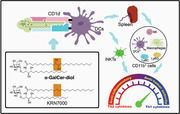

## 1 Introduction

Helper T cell 1 (Th1)‐polarizers have the potential to be applied as vaccine adjuvants to treat a large array of diseases that present with immunological disorders, including cancers and infections. KRN7000, well‐studied *α*‐galactosylceramide (*α*‐GalCer) glycolipid, is a promising Th1‐polarizer; however, its development as an agonist to boost immunity is undermined by its simultaneous induction of both Th1‐ and helper T cell 2 (Th2)‐type cytokines with little selectivity.^[^ ^1^, ^2^ ^]^ This has led to efforts to identify additional KRN7000 analogs that are able to selectively activate either the Th1 or Th2 response. Specifically, many KRN7000 analogs have been designed and synthesized in recent years due to their potent ability to drive Th1‐ or Th2‐type cytokine production,^[^ ^3^, ^4^, ^5^, ^6^, ^7^, ^8^ ^]^ but few candidates have proven to be therapeutically feasible for controlling cytokine profiles. To this end, there is a strong need for additional potent and selective Th1‐priming agonists.

The mode of action of *α*‐GalCer is largely attributed to its strong binding to cluster of differentiation 1d (CD1d), a member of the CD1 family of glycoproteins expressed on the plasma membrane of antigen‐presenting cells (APCs), such as dendritic cells (DCs).^[^ ^9^ ^]^ When the *α*‐GalCer‐CD1d complex is presented to the semi‐invariant T‐cell receptor (TCR) on invariant natural killer T cells (iNKTs), a ternary complex is formed, leading to the secretion of various cytokines.^[^ ^10^ ^]^ As a major feature of activated iNKT cells, these cells are able to secrete different sets of cytokines under differential priming states, such as Th1, Th2, and helper T cell 17 (Th17) subtype priming.^[^ ^11^ ^]^ Mounting evidence has revealed that structural modifications of KRN7000 could greatly influence the Th1 versus Th2 responses (Table S1, Supporting Information). However, no fundamental principles underlying the skew of cytokine production toward either the Th1 or Th2 type have been identified. Nonetheless, enhanced interaction between CD1d and glycolipids, though a stabilized trimolecular complex, has been found to lead to a Th1‐biased polarization.^[^ ^12^, ^13^, ^14^ ^]^ Under this premise, diverse modifications of KRN7000 have been proposed to enhance the binding affinity between CD1d and glycolipids, or between CD1d/glycolipid and TCR. For example, changes in the acyl chain, sphingosine chain, glycosidic bond, or the hydroxyl groups on the pyranose ring, could alter the stability of the CD1d/glycolipid binary or the association between the binary and TCR to favor the production of interferon‐*γ* (IFN‐*γ*) (indicative of Th1 response) over that of interleukin 4 (IL‐4) (representative of Th2 response). ^[^ ^6^, ^7^, ^8^, ^10^, ^12^, ^13^, ^15^, ^16^, ^17^, ^18^, ^19^ ^]^ A selection of observations regarding Th1‐biased modifications are shown in Scheme S1 (Supporting Information). However, paradoxical results and the low stability and reactivity of *α*‐GalCer analogs have limited their use as therapeutics for cancer, infection, or autoimmune diseases.^[^ ^4^, ^12^, ^20^, ^21^, ^22^, ^23^ ^]^ As such, it remains a challenge to design *α*‐GalCer analogs able to induce Th1‐ or Th2‐biased cytokine production.

As the predominant type of APCs, DCs have been extensively studied in the CD1d‐restricted and TCR‐mediated activation of iNKT cells, triggered by *α*‐GalCer analogs. In contrast, much less is known about the contribution of other types of APCs in promoting iNKT cell activation. In addition, as a distinct population of CD1d‐restricted T‐lymphocytes, iNKT cells are able to polarize the immune response and interact with other types of immune cells, providing iNKT cells with outstanding properties for immunotherapies. Currently, the source of Th1‐type cytokines, such as IFN‐*γ*, is exclusively attributed to iNKT activation. However, the direct/indirect effects of *α*‐GalCer analogs on various types of immune cells (even CD1d‐independent mechanisms) and the outcomes downstream of iNKT activation should also be taken into account when assessing the molecular basis of variant analog function. In the current study, we designed a new *α*‐GalCer analog, *α*‐GalCer‐diol, by introducing hydroxyl groups to the proper sites of acyl chain based on computational modeling, which were expected to form hydrogen bonds with polar residues in the A’ pocket of CD1d. Our results revealed that *α*‐GalCer‐diol had a higher affinity for CD1d and ultimately elicited a greater IFN‐*γ*‐biased immune response ex vivo and in vivo than KRN7000. Importantly, we also uncovered a new mechanism underlying the *α*‐GalCer‐diol‐induced Th1‐type response, different from that of KRN7000. The current study opens a new avenue for identifying selective *α*‐GalCer analogs to enable customized therapeutics.

## 2 Results and Discussion

### 2.1 Molecular Design

The CD1d/glycolipid interaction has been reported to bias cytokine secretion in favor of Th1‐type cytokines.^[^ ^12^, ^13^ ^]^ Structural analysis revealed that there are primarily polar residues (CYS‐12, GLN‐14, SER‐28, TYR‐73, and CYS‐168, as shown in Scheme S2, Supporting Information) in the A’ pocket of CD1d, with the exception of previously reported hydrophobic and aryl residues.^[^ ^4^, ^12^ ^]^ Thus, new glycolipids were formed by introducing polar hydroxyl groups at the proper positions of the acyl chain, and the binding interaction between the designed compounds and CD1d was modeled with Surflex‐Dock in SYBYL software (Tripos Inc., St. Louis, MO).

**Scheme 1:**
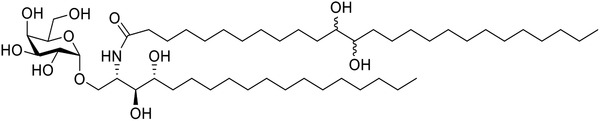
Structure of a new glycolipid, *α*‐GalCer‐diol.

Based on structural analyses of the CD1d/glycolipid binary complex, hydroxyl groups were introduced on the 9^–^14th and 22–24th positions of the acyl chain (Scheme S3, Supporting Information) to identify sites with steady affinity toward the polar residues GLN‐14, SER‐28, TYR‐73, or CYS‐168. Docking calculations indicated that introducing hydroxyls on the 12th and 13th positions of the acyl chain (Scheme S3, Supporting Information) resulted in a higher TotalScore than that of KRN7000. Compared to KRN7000, additional hydrogen bond interactions were observed between GLN‐14, SER‐28, or TYR‐73 and the added hydroxyl groups of the designed glycolipids (**Scheme** **1**), supporting a stronger CD1d binding ability for the designed glycolipids. In addition, the four designed diastereomers (Scheme S4, Supporting Information) had comparable TotalScores and binding patterns with CD1d, suggesting that it would be reasonable to use racemic *α*‐GalCer‐diol in subsequent experiments. To verify our predictions, *α*‐GalCer‐diol was synthesized and its activity was evaluated. The total synthesis of *α*‐GalCer‐diol (**1**, Scheme S6, Supporting Information) was accomplished in nine chemical reaction steps, achieving an overall yield of 14.3% using commercially available methyl 12‐hydroxydodecanoate (**2**, Scheme S5, Supporting Information), phytosphingosine (**7**, Scheme S6, Supporting Information), and benzylated isopropyl‐*β*‐*D*‐thiogalactopyranoside (IPTG) (**10**, Scheme S6, Supporting Information) as starting materials. The key reactions applied in this strategy were Dess–Martin oxidation, olefination, Sharpless dihydroxylation, and stereoselective glycosylation. Detailed synthetic procedures were presented in the Supporting Information.

### 2.2 Enhanced Interaction between *α*‐GalCer‐diol and CD1d

**Scheme 2:**
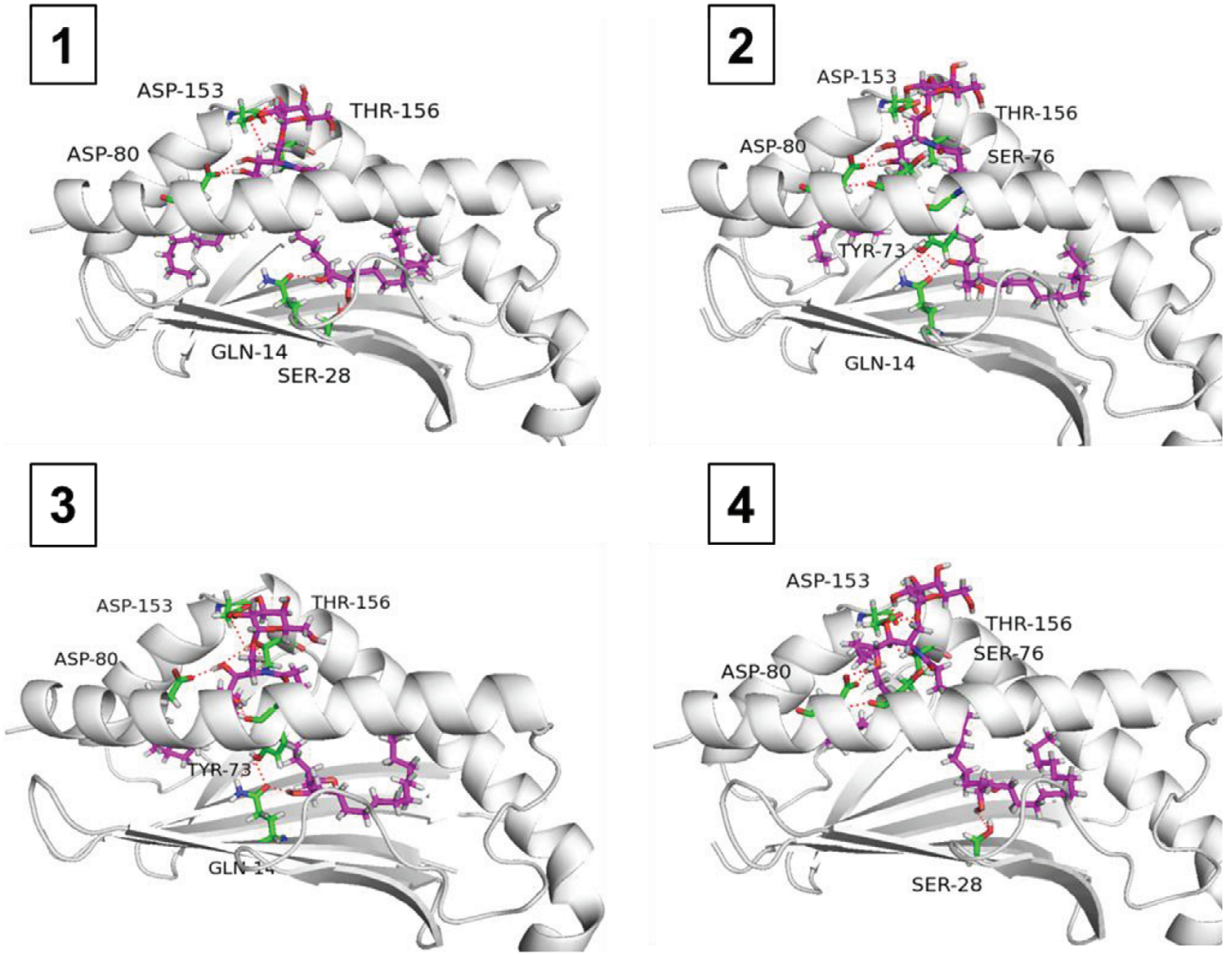
Structures of the binary complex formed by CD1d and four diastereomers of *α*‐GalCer‐diol. For CD1d, the carbon of the acyl chain and key residues are colored white and green, respectively. *α*‐GalCer‐diol is denoted by cyan, and the hydrogen bonds formed between CD1d and *α*‐GalCer‐diol are shown in red.

**Figure 1:**
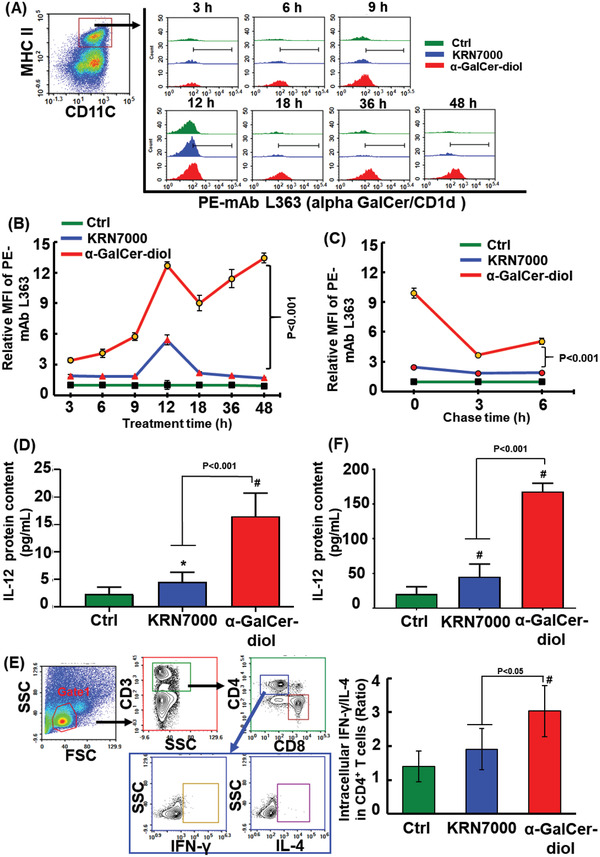
Enhanced interaction of *α*‐GalCer‐diol with CD1d. A) BMDCs were incubated with 300 × 10^−9^ m *α*‐GalCer‐diol or KRN7000 for different time periods. Thereafter, 2 × 10^4^ CD11c^+^MHC II^+^ BMDCs in each sample (the left panel) were gated to assess the MFI of the mCD1d/a‐GalCer complex using mAb L363. Histogram illustrations show L363 staining of cells upon treatment with *α*‐GalCer‐diol (red) and KRN7000 (blue). B) The MFI of PE‐mAb L363 staining in BMDCs following *α*‐GalCer‐diol or KRN7000 treatment after 3, 6, 9, 12, 18, 36, and 48 h relative to the untreated control group. C) The relative MFI of L363 staining in BMDCs at each chase time (3 and 6 h) after pretreatment with *α*‐GalCer‐diol or KRN7000 for 12 h. D) Levels of IL‐12 protein in the supernatants collected from BMDCs (1 × 10^6^/well) treated with 1.26 × 10^−9^ m *α*‐GalCer‐diol or KRN7000 for 24 h. E) Determination of intracellular IFN‐*γ* and IL‐4 levels in CD4^+^ T cells. BMDCs were collected after treatment with 1.26 × 10^−9^ m *α*‐GalCer‐diol or KRN7000 for 24 h. Spleen cells were isolated from mice pretreated with 4 nmol *α*‐GalCer‐diol or KRN7000 for 72 h. Thereafter, spleen cells (1 × 10^6^/well) were coincubated with BMDCs (1 × 10^5^/well) for 6 h, and the levels of intracellular IFN‐*γ* and IL‐4 in gated lymphocytes (5 × 10^4^ cells, in the red circle, in the left panel) were analyzed by FACS. The ratio of IFN‐*γ* ^+^ to IL‐4^+^ cells in CD4^+^ T cells is shown in the right panel. F) IL‐12 protein content in the supernatants was assayed after 48 h of coincubation. Asterisk (*) indicates *P *< 0.05, and the pound sign (#) denotes *P *< 0.001, compared to the control group (*n* = 5).

CD1d, a Type II CD1 molecule that is predominantly expressed by APCs,^[^ ^24^, ^25^ ^]^ adopts an MHC class I‐like structure with a hydrophobic antigen‐binding cleft that harbors deep pockets well‐suited for the binding of lipid antigens. Thus, diverse APCs have been demonstrated to present lipid antigens to iNKT cells, and DCs represent the major APCs presenting glycolipids.^[^ ^26^, ^27^ ^]^ To verify the above simulation data on the interaction between *α*‐GalCer‐diol and CD1d (Scheme S3, Supporting Information; and **Scheme** **2**), the presentation of *α*‐GalCer‐diol and subsequent interaction with CD1d were assayed in primary cultured bone marrow‐derived dendritic cells (BMDCs) in comparison to KRN7000 using L363 monoclonal antibody (mAb) staining, an established method to specifically identify the complex formed between glycolipids and CD1d.^[^ ^28^, ^29^ ^]^ As shown in **Figure** **1**A, fluorescence‐activated cell sorting (FACS) analysis identified positive L363 staining in CD11c^+^MHCII^+^ BMDCs, and much more L363‐positive cells were observed in BMDCs upon the addition of *α*‐GalCer‐diol compared to KRN7000 over the time course. As reflected by the mean fluorescence intensity (MFI), the formation of the binary *α*‐GalCer‐diol‐CD1d complex was observed to be much greater than that of KRN7000 over time (Figure 1B, *P* < 0.001); an obvious peak was only found for KRN7000 at 12 h (Figure 1B). In support of these data, similar results were also observed in DC2.4 cells, an immortalized line of DCs (Figure S1, Supporting Information, *P* < 0.001). Furthermore, pulse‐chase analysis was performed to assess the dynamic change in CD1d‐glycolipid complexes. After preincubation with *α*‐GalCer‐diol or KRN7000 for 12 h, BMDCs were recultured for 3 and 6 h in the absence of glycolipid treatment. As shown in Figure 1C, compared to the rather low level of L363 staining for the KRN7000‐CD1d complex, the MFI value was much greater for the *α*‐GalCer‐diol‐CD1d complex, even after a decrease at 6 h (*P* < 0.001). This suggested a more tight and steady formation of the *α*‐GalCer‐diol‐CD1d complex on the cell membrane than that of KRN7000.

DCs are also activated by glycolipid presentation, characterized by induction of the surrogate cytokine IL‐12.^[^ ^30^, ^31^, ^32^ ^]^ In this context, IL‐12 content was measured in the supernatant collected from BMDCs upon *α*‐GalCer‐diol or KRN7000 treatment. Remarkably, *α*‐GalCer‐diol increased IL‐12 production in BMDCs by ≈7 fold relative to the untreated control, whereas KRN7000‐treated cells exhibited only a 2‐fold increase in IL‐12 levels (Figure 1D, *P *< 0.001), indicating that more DCs were activated upon *α*‐GalCer‐diol treatment. As a crucial organ for antigen presentation, spleen cells are commonly employed as an ex vivo model to test the efficacy of antigen presentation and the interaction between different types of cells.^[^ ^33^ ^]^ Thus, glycolipid‐laden BMDCs were cocultured with spleen cells from mice challenged by corresponding *α*‐GalCer‐diol or KRN7000, respectively, following an established method.^[^ ^33^, ^34^ ^]^ To determine the efficacy of antigen presentation, the intracellular expression of IFN‐*γ* and IL‐4 in CD4^+^ cells was analyzed by FACS (Figure 1E, the left panel). After calculating the ratio of intracellular IFN‐*γ* to IL‐4, the ratio was found to be increased by 37% in the CD4^+^ subpopulation of KRN7000‐exposed cocultured cells, and increased by 117% in the CD4^+^ subpopulation of *α*‐GalCer‐diol‐exposed cells, relative to the untreated control (Figure 1E, *P* < 0.05, the right panel). This suggested a greater Th1‐biased polarization of CD4^+^ cells in BMDCs following *α*‐GalCer‐diol treatment than KRN7000 treatment. Moreover, as shown in Figure 1F, IL‐12 content was increased by 2.54‐fold in the coculture medium upon KRN7000 treatment, and was increased by 4.69‐fold upon *α*‐GalCer‐diol treatment relative to the untreated control (Figure 1F, *P* < 0.001). This indicated a reinforced activation of DCs following antigen presentation of *α*‐GalCer‐diol. Collectively, these data revealed that *α*‐GalCer‐diol exhibited a strong ability to bind CD1d and was relatively more potent to activate DCs than KRN7000.

### 2.3 Potent Th1 Cytokine Response Induced by *α*‐GalCer‐diol

Motivated by the above findings, we further addressed our prediction that increasing the CD1d affinity of glycolipids would promote a Th1‐type cytokine response. To assess this, IFN‐*γ* and IL‐4 concentrations were assayed in the sera of mice following the administration of *α*‐GalCer‐diol and KRN7000, respectively, based on a previously established approach.^[^ ^24^, ^29^ ^]^ Figure S2A (Supporting Information) showed a significant time‐dependent induction of IFN‐*γ* production with a pronounced peak around 12 h for both glycolipids (*P* < 0.05). Similar to previous reports,^[^ ^35^, ^36^, ^37^ ^]^ KRN7000 provoked a rapid burst of IL‐4 production around 3 h (Figure S2B, Supporting Information, *P* < 0.05). Analogously, *α*‐GalCer‐diol evoked a similar pattern of IL‐4 secretion (Figure S2B, Supporting Information, *P* < 0.05). Furthermore, the production (as determined by the area under curve, AUC, as established previously)^[^ ^18^ ^]^ of Th1‐type cytokines (IFN‐*γ*, IL‐2, IL‐12, tumor necrosis factor‐*α*, and granulocyte‐macrophage colony‐stimulating factor (GM‐CSF)) and Th2‐type cytokines (IL‐4, IL‐5, and IL‐10) were assessed in mice upon KNR7000 and *α*‐GalCer‐diol administration. As shown in Figure S2C (Supporting Information), *α*‐GalCer‐diol greatly stimulated the Th1‐type cytokine response, similar to KRN7000, as evidenced by the much greater value of AUC corresponding to Th1‐type cytokines than that of Th2 cytokines. Therefore, these results confirmed the marked capability of *α*‐GalCer‐diol to stimulate Th1‐biased immune responses.

### 2.4 Greater Efficacy of *α*‐GalCer‐diol in Stimulating iNKT Activation

**Figure 2:**
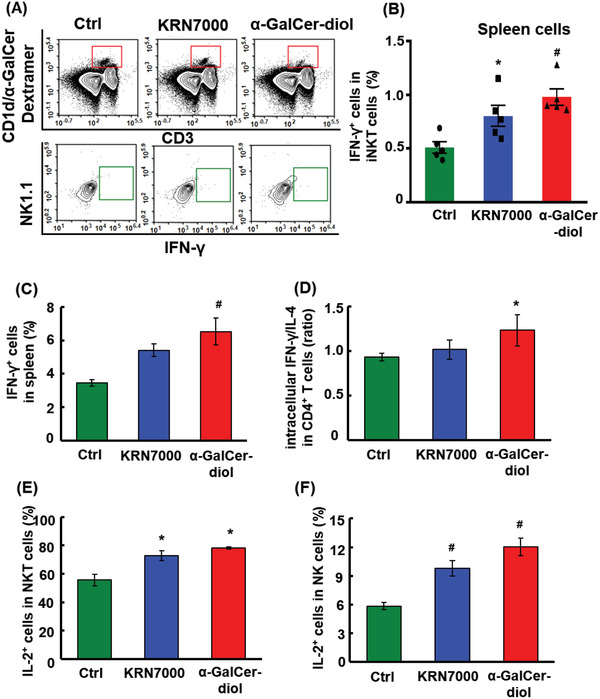
Higher efficacy of *α*‐GalCer‐diol in stimulating iNKT activation. A) Isolated spleen cells were subjected to subpopulation sorting dependent on CD3, mCD1d/*α*‐GalCer Dextramer, and NK1.1 staining to identify activated iNKT cells by FACS. B) The percentage of IFN‐*γ* ^+^ iNKT cells in spleen cells under different treatments. C) The percentage of IFN‐*γ* ^+^ cells in mouse splenocytes following glycolipid stimulation for 6 h ex vivo. D) The ratio of intracellular IFN‐*γ* to IL‐4 in CD4^+^ T cells in splenocytes isolated from mice challenged with glycolipids for 6 h. E) The percentage of IL‐2^+^ cells in NKT cells and F) NK cells in isolated splenocytes after glycolipid stimulation for 6 h ex vivo. Asterisk (*) indicates *P *< 0.05, and the pound sign (#) denotes *P *< 0.001, compared to the control group (*n* = 5).

With respect to the Th1‐type cytokine response, IFN‐*γ* induction is an outstanding feature.^[^ ^24^, ^38^ ^]^ Because the spleen is the primary site of NKT localization,^[^ ^39^, ^40^, ^41^ ^]^ the direct interaction between glycolipids and spleen cells was assessed ex vivo. First, we specifically identified iNKT cells using a phycoerythrin (PE)‐conjugated CD1d tetramer,^[^ ^27^, ^29^ ^]^ which enables identification of iNKT cells in response to antigen presentation characterized by IFN‐*γ* production (**Figure** **2**A). As shown in Figure 2B, iNKT lymphocytes (distinguished as CD3^+^NK1.1^+^mCD1d^+^ tetramer^+^) were highly activated upon *α*‐GalCer‐diol treatment, as demonstrated by a 93% increase in IFN‐*γ* ^+^ iNKT cells versus a 57% increase upon KRN7000 treatment, compared to the untreated control (*P* < 0.05). Meanwhile, intracellular staining of IFN‐*γ* in total spleen cells after incubation with *α*‐GalCer‐diol or KRN7000 showed that the percentage of overall IFN‐*γ* ^+^ cells was increased, especially in the *α*‐GalCer‐diol‐treated group, which had a 1.7‐fold increase (*P* < 0.001), compared to that in the control group (Figure 2C). Upon assessing the intracellular levels of IFN‐*γ* and IL‐4 among CD4^+^ T cells, a significant increase in the ratio of IFN‐*γ* to IL‐4 was observed following *α*‐GalCer‐diol treatment, with a 30% increase relative to the untreated control (*P* < 0.05), while only a very slight increase in KRN7000‐treated cells (Figure 2D).

To verify the differentially activated priming states of CD4^+^ T cells, a surrogate cytokine, IL‐2, was assessed in the spleen cells of mice following different treatments. As previously reported, IL‐2 is crucial for the maintenance of functional T cells in an autocrine manner,^[^ ^42^ ^]^ and is also important in modulating other cell types, such as NK cells and innate lymphoid cells.^[^ ^43^, ^44^ ^]^ Consistent with the increase of IFN‐*γ* ^+^ cells observed in whole spleen cells (Figure 2B–D), the intracellular concentration of IL‐2 was greatly elevated in response to KRN7000 and *α*‐GalCer‐diol treatment, especially the latter, as evidenced by the increased percentage of IL‐2^+^ cells in the NKT and even the NK subpopulations (Figure 2E,F, *P* < 0.05). These data indicated a higher level of IFN‐*γ* ^+^ iNKT cell activation upon the administration of *α*‐GalCer‐diol than KRN7000. Our findings thereby signified a greater Th1‐biased response induced by *α*‐GalCer‐diol through DC‐conducted CD1d‐restricted and TCR‐mediated activation of iNKT cells, and implicated other types of immune cells in promoting Th1 responses either downstream of DC and iNKT activation or by a direct effect on those cells.

### 2.5 Greater Activation of CD45^+^CD11b^+^ Leukocytes toward the Th1 Type Response Upon *α*‐GalCer‐diol Treatment

**Figure 3:**
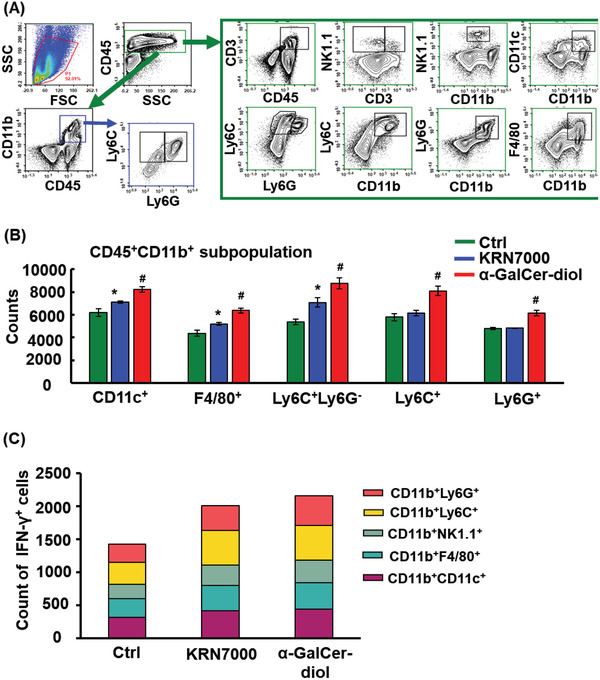
Greater activation of CD45^+^CD11b^+^ leukocytes toward the Th1‐type response upon *α*‐GalCer‐diol treatment. Following intravenous (I.V.) administration for 24 h, the spleens of glycolipid treated mice were dissected for the preparation of single cell suspensions and FACS analysis. A) Graded analysis of various subpopulations from total gated cells (5 × 10^5^ cells) as follows: CD3^+^ T cells, CD3^+^NK1.1^+^ subpopulation representative of NKT cells, CD11b^+^NK1.1^+^ subpopulation suggestive of NK cells, CD11b^+^CD11c^+^ subpopulation representative of dendritic cells, CD11b^+^F4/80^+^ denoting macrophages, CD11b^+^Ly6G^+^ subpopulation indicative of neutrophils, CD11b^+^Ly6C^+^ subpopulation marking mononuclear macrophages, and CD11b^+^Ly6G^−^Ly6C^+^ subpopulation representative of mononuclear leukocytes. B) Quantification of diverse subpopulations of CD45^+^ CD11b^+^ cells. C) Count of IFN‐*γ* ^+^ cells in each CD11b^+^ subtype splenocytes isolated from mice following glycolipid treatment for 6 h. Asterisk (*) indicates *P *< 0.05, and the pound sign (#) denotes *P *< 0.001, compared to the control group (*n* = 5).

Inspired by previous reports, cytokine levels in the sera of mice following inducer treatment are able to largely characterize the overall pool of cytokines produced by a variety of cell types through direct or indirect mechanisms.^[^ ^45^, ^46^ ^]^ However, there are currently few known sources of IFN‐*γ* production following glycolipid treatment, such as iNKT cells. Therefore, we investigated the molecular mechanisms underlying the stimulation of Th1‐type cytokine production by *α*‐GalCer‐diol. This may be dependent on or independent of the CD1d‐restricted and TCR‐mediated activation of iNKT cells. Additionally, we attempted to scrutinize potential sources contributing cells to Th1 responses that were differentially induced by *α*‐GalCer‐diol and KRN7000. FACS analysis revealed the expansion of leukocytes, as demonstrated by increased proportions of CD45^+^CD54^+^ cells (a marker to identify leukocytes),^[^ ^47^ ^]^ in the spleens of treated mice compared to those of untreated mice (Figure S3, Supporting Information). Furthermore, we interrogated the individual subpopulations within CD45^+^ leukocytes. Intriguingly, the CD11b^+^ subpopulation was greatly enriched in the spleens of mice treated with KRN7000 and *α*‐GalCer‐diol, especially the latter, as determined by the graded gating of CD45^+^ leukocytes using FACS (**Figure** **3**A). As shown in Figure 3B, quantitative analysis of the CD45^+^CD11b^+^ subpopulation revealed that the numbers of CD11c^+^ cells and F4/80^+^ cells were increased upon glycolipid treatment compared to those in the control group, with a 32.8% increase in CD11c^+^ cells and 45.5% increase in F4/80^+^ cells following *α*‐GalCer‐diol treatment (*P* < 0.001), in contrast to a 14.6% increase in CD11c^+^ cells and 18.6% increase in F4/80^+^ cells following KRN7000 treatment (*P* < 0.05). For CD11b^+^Ly6C^+^ and CD11b^+^Ly6G^+^ cells, *α*‐GalCer‐diol treatment led to an ≈39.6% and 28.2% increase (*P* < 0.001), respectively, relative to the control group, showing stronger ability than KRN7000 (Figure 3B, *P* < 0.05). Similarly, Ly6C^+^Ly6G^−^ cells were increased by 62.8% (*P* < 0.001) and 31.8% (*P* < 0.05) upon *α*‐GalCer‐diol and KRN7000 treatment, respectively, compared to the control group.

Next, we aimed to determine if increased CD11b^+^ cells also contributed to the reinforced Th1 responses induced by glycolipids. For this purpose, we investigated the Th1‐priming polarization of CD11b^+^ cells by measuring the intracellular IFN‐*γ* level. As shown in Figure 3C, elevated IFN‐*γ* levels were found in CD11b^+^ cells in the spleens of treated mice, particularly with *α*‐GalCer‐diol treatment, demonstrating reinforced polarization of the Th1‐type response of CD11b^+^ cells upon exposure to *α*‐GalCer‐diol. Moreover, increased IFN‐*γ* levels were found in all subpopulations of CD11b^+^ cells, including CD11c, F4/80, Ly6C, Ly6G, and NK1.1 (Figure 3C). Given that CD1d is expressed by many hematopoietic cells,^[^ ^39^, ^48^ ^]^ it would be of great interest to clarify whether the increase in IFN‐*γ* levels was due to altered CD1d expression in CD11b^+^ cells following *α*‐GalCer‐diol treatment. Thus, we assessed CD1d expression in each cell subpopulation in more details. Although the content of CD1d in each CD45^+^CD11b^+^ subpopulation varied, KRN7000 and *α*‐GalCer‐diol treatment did not alter CD1d expression (Figure S4, Supporting Information). Collectively, these data indicated that *α*‐GalCer‐diol stimulated the expansion and Th1‐priming state of CD11b^+^ leukocytes through some currently unknown mechanisms, which may be dependent on or independent of DC‐conducted CD1d‐restricted and TCR‐mediated iNKT cell activation.

### 2.6 *α*‐GalCer‐diol Promoted IFN‐*γ* Production in Monocytes/Macrophages Independent of DC‐Mediated CD1d‐Restricted Activation of iNKT Cells

**Figure 4:**
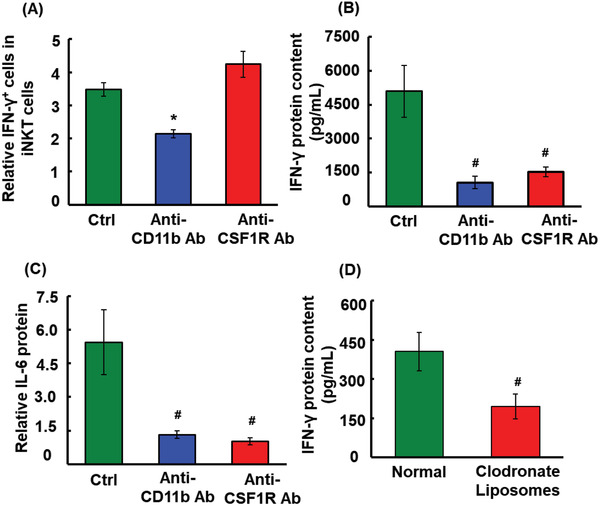
IFN‐*γ* and IL‐6 production in CD11b^+^ leukocytes in response to *α*‐GalCer‐diol treatment. Following intraperitoneally (I.P.) administration of 5 mg kg^−1^ body weight anti‐CD11b antibody or anti‐CSF‐1R antibody for 24 h, mice were further challenged by 4 nmol *α*‐GalCer‐diol for another 24 h, followed by serum and spleen collection. A) Quantitative analysis of the percentage of IFN‐*γ* ^+^ iNKT cells in splenocytes from mice following anti‐CD11b antibody or anti‐CSF‐1R antibody treatment relative to the untreated control. B) IFN‐*γ* protein levels in each group. C) Serum concentrations of IL‐6 in each group. D) Following I.V. administration of 5 mg kg^−1^ body weight clodronate liposomes for 48 h, mice were further challenged with 4 nmol *α*‐GalCer‐diol for an additional 24 h, followed by the detection of serum IFN‐*γ* in each group. Asterisk (*) indicates *P *< 0.05, and the pound sign (#) denotes *P *< 0.001, compared to the control group (*n* = 5).

To determine the mechanisms underlying the cellular source of IFN‐*γ* production by CD11b^+^ cells, an anti‐CD11b antibody was used to deplete CD11b^+^ cells by neutralizing CD11b in mice treated with *α*‐GalCer‐diol, following an established protocol.^[^ ^49^ ^]^ As shown in Figure S5A,B (Supporting Information), CD11b antibody treatment was found to be fairly efficient in neutralizing and depleting endogenous CD11b^+^ cells, including DCs (CD45^+^CD11b^+^CD11c^+^), monocytes/macrophages (CD45^+^CD11b^+^F4/80^+^), NK (CD45^+^CD11b^+^NK1.1^+^), neutrophils (CD45^+^CD11b^+^Ly6G^+^), mononuclear macrophages (CD45^+^CD11b^+^Ly6C^+^), and mononuclear leukocytes (CD45^+^CD11b^+^Ly6G^−^Ly6C^+^), as determined by FACS analysis. As a result, the IFN‐*γ* ^+^ subpopulation of iNKT cells was reduced by 31% in the spleens of *α*‐GalCer‐diol‐treated mice relative to the untreated control (**Figure** **4**A, *P* < 0.05), leading to an 80% reduction in serum IFN‐*γ* concentration (Figure 4B, *P* < 0.001). This finding suggested that CD11b^+^ cells primarily account for *α*‐GalCer‐diol presentation and subsequent iNKT cell activation, and also implied the possibility of IFN‐*γ* production from CD11b^+^ cells independent of iNKT cells.

As previously established,^[^ ^50^ ^]^ the largest proportion of CD11b^+^ cells consisted of monocytes/macrophages, DCs, and other minor immune cells, as shown in Figure 3B,C. In this context, to isolate the contribution of monocytes/macrophages in activating iNKT cells and inducing the overall serum IFN‐*γ* levels, a neutralization/depletion experiment was performed using an antibody against colony stimulating factor 1 receptor (CSF‐1R) (Figure S5A,B, Supporting Information). As previously described,^[^ ^51^, ^52^ ^]^ CSF‐1R, also known as macrophage colony‐stimulating factor receptor, is predominantly expressed in monocytes/macrophages and plays a crucial role in regulating the survival, growth, and differentiation of myeloid lineage cells. Surprisingly, depletion of monocytes/macrophages did not reduce the proportion of the IFN‐*γ* ^+^ subpopulation among iNKT cells (Figure 4A); however, the overall serum IFN‐*γ* concentration was reduced by 70%, compared to the untreated control (Figure 4B, *P *< 0.001). In support of this observation, similar data were demonstrated for another pro‐inflammatory cytokine, IL‐6 (Figure 4C, *P* < 0.001). To confirm this discovery, clodronate liposomes were pre‐administered to *α*‐GalCer‐diol‐treated mice to deplete endogenous macrophages.^[^ ^53^ ^]^ Considerable depletion of endogenous monocytes/macrophages, including CD11b^+^F4/80^+^, CD11b^+^CD169^+^, and CD11b^+^CD169^+^F4/80^+^ subtypes, was verified in the spleens of *α*‐GalCer‐diol‐treated mice upon clodronate liposome administration (Figure S6, Supporting Information, *P* < 0.001). As a consequence, IFN‐*γ* content in sera was decreased by 52% relative to the untreated group (Figure 4D, *P* < 0.001). Together, these results suggested that monocytes/macrophages were an important source of *α*‐GalCer‐diol‐induced IFN‐*γ* production independent of iNKT cells.

### 2.7 *α*‐GalCer‐diol Showed Higher Antitumor Activity

**Figure 5:**
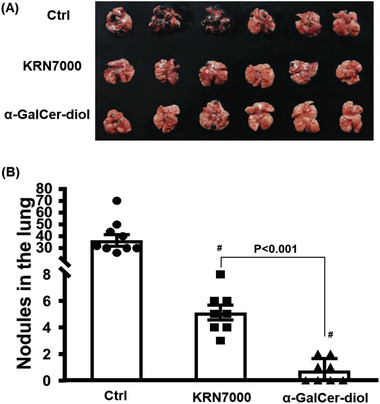
*α*‐GalCer‐diol showed higher antitumor activity than KRN7000. A) Representative image of lungs with tumor nodules (in dark color) from mice following KRN7000 or *α*‐GalCer‐diol treatment (*n* = 6). B) Quantification of the number of tumor nodules in each group. Asterisk (*) indicates *P *< 0.05, and the pound sign (#) denotes *P *< 0.001, compared to the control group (*n* = 8–9).

Downstream of elevated Th1‐biased responses, one would expect to see a reinforced capability to combat intruding parasites and cancer cells.^[^ ^54^, ^55^ ^]^ As described above, our data revealed that *α*‐GalCer‐diol triggered greater Th1‐biased responses than KRN7000 by stimulating DC‐mediated CD1d‐restricted activation of iNKT cells and activating monocytes/macrophages through either a direct or indirect mechanism due to iNKT and DC activation. These changes would certainly endow *α*‐GalCer‐diol enhanced anticancer activities. In this context, the anticancer capability of *α*‐GalCer‐diol was assessed using an established B16‐F10 melanoma model.^[^ ^56^ ^]^ Compared to untreated mice, metastatic nodules in the lung were noticeably repressed by both *α*‐GalCer‐diol and KRN7000 treatment, and *α*‐GalCer‐diol exhibited a much stronger antitumor activity with ≈70% reduction in nodule numbers in the lung compared to those of KRN7000‐treated mice (**Figure** **5**A,B, *P* < 0.001). Therefore, these antitumor data signified that the remarkable Th1‐biased response induced by *α*‐GalCer‐diol was capably of restraining tumor progression.

## 3 Conclusions

Although immunotherapy is a promising strategy to combat a diverse array of disorders, including cancers and viral infections, nonselective production of Th2‐type cytokine profiles limits the application of most Th1‐biased agonists. In the current study, we utilized machine learning to predict the interactions between glycolipids, CD1d, and TCR, leading to the addition of two hydroxyl groups to the acyl chain of KRN7000 to produce a new type of agonist, *α*‐GalCer‐diol. Ex vivo and in vivo results revealed a greater potency of *α*‐GalCer‐diol in promoting a Th1‐type cytokine profile than the most widely studied agonist, KRN7000. Regarding the molecular mechanisms, similar to KRN700, *α*‐GalCer‐diol boosted Th1‐biased responses through CD1d‐restricted and TCR‐mediated activation of iNKT cells. However, unlike KRN7000, *α*‐GalCer‐diol was able to target CD11b^+^ cells to drive their expansion and Th1‐biased polarization. These pronounced features conferred *α*‐GalCer‐diol with an enhanced capability to inhibit tumor progression. This study represents a new strategy to design Th1‐biased *α*‐GalCer analogs for antitumor immunotherapy.

## 4 Experimental Section

##### Molecular Docking

Surflex‐Dock was employed to model the interaction of the designed *α*‐GalCer‐diol and CD1d using SYBYL 8.0 software (Tripos Inc., St. Louis, MO). The crystal structure of CD1d was obtained from the Protein Data Bank (PDB code: 3HE6; www.rcsb.org). A chain of 3HE6 was used to study the interaction between glycolipids and CD1d. The initial structure of KRN7000 was extracted from 3HE6, and the structures of the designed glycolipids were derived from KRN7000. Water was removed from the PDB file before docking, and all hydrogen atoms were added. Meanwhile, Gasteiger–Hückel atomic charge was added to the ligands and proteins. To limit the effect of initial ligand conformation on docking results, three starting conformations of each ligand, obtained from ligand energy minimization with Max Iterations set to 0, 100, or 1000, were applied, respectively. Prior to docking, Protomol was generated with Threshold 0.5 and Bloat 10.0 Å. Parameters of Surflex‐Docking were set to the default values, except for the use of the extracted ligand of KRN7000 as a reference molecule. To verify the feasibility of the parameters, KRN7000 was first docked onto the binding site. The root‐mean‐square deviation of KRN7000 was 1.59 Å, indicating that the parameters were reasonable. The TotalScore of each ligand was used to determine its binding ability toward CD1d, followed by a computational structure analysis using PyMol software (https://pymol.org/2/).

*Chemical Synthesis of α‐GalCer‐diol*: All chemicals used in this study were purchased from commercial suppliers. All moisture‐sensitive reactions were carried out under a nitrogen atmosphere using flame‐dried glassware. Anhydrous solvents were obtained by standard procedures. ^1^H NMR and ^13^C NMR spectra were recorded with a Bruker AVANCE‐III 400 MHz spectrometer (100 MHz for ^13^C NMR). Chemical shifts were reported in ppm with respect to internal tetramethylsilane. Mass spectra data were acquired with electrospray ionization on a Bruker micro‐ time‐of‐flight (TOF) Q II mass spectrometer or matrix‐assisted laser desorption/ionization time‐of‐flight (MALDI‐TOF) mass spectra on a Kratos MALDI II spectrometer. Thin‐layer chromatography was performed on silica gel HF_254_ through detection by charring with 20% v/v H_2_SO_4_ in methanol. Column chromatography was performed on a column of silica gel (100–200 mesh). Solutions were concentrated at low temperatures (below 50 °C).

The detailed procedure for the synthesis of *α*‐GalCer‐diol and the corresponding NMR assessments are described in the Supporting Information. The target compound, *α*‐GalCer‐diol, and the reference compound, KRN7000, were diluted in dimethyl sulfoxide (in vitro pure, Solarbio Inc., Beijing, China) and warmed in a water bath at ≈40 °C to ensure full dissolution prior to experimentation.

##### Animal Use

All animal care and experimental procedures were approved by the Animal Ethics Committee at the Research Center for Eco‐Environmental Sciences, Chinese Academy of Sciences (CAS). C57BL/6 wild‐type male mice (7 weeks old and with body weights ≈22–25 g) were purchased from the Vital River Laboratory Animal Technology Co. Ltd, Beijing, China. All animals were housed under standard specific pathogen‐free conditions and randomly divided into experimental groups.

##### Systemic Cytokine Profile Assay

For cytokine profile analysis, mice were administered 4 nmol (in 100 µL phosphate buffered saline (PBS)) *α*‐GalCer‐diol or KRN7000 through tail vein I.V. injection. At each time point, blood was collected and the concentrations of IL‐4 and IFN‐*γ* were quantified using commercial ELISA kits (NeoBioscience, Shenzhen, China). Other cytokine profiles were determined using the Bio‐Plex Pro Mouse Cytokine Th1/Th2 Assay package (Biorad, M6000003J7) with a Luminex 200 platform and XPONET 3.1 software.

##### Cell Lines and Primary Cell Culture

B6‐derived DC line DC2.4 was purchased from the Shanghai Cell Bank of the Type Culture Collection of the Chinese Academy of Sciences. The B16‐F10 mouse melanoma cell line was generously provided by Dr. Min Fang (Institute of Microbiology, CAS, Beijing, China). Single cell suspension of mouse spleen cells was prepared as previously described.^[^ ^57^ ^]^ Recombinant murine IL‐2 was added to the splenocyte culture medium to maintain T‐cell proliferation (≥5 × 10^6^ units mg^−1^, Pepro Tech, Inc. USA) at 20 U mL^−1^. BMDCs were prepared and cultured as follows. Briefly, bone marrow cells were flushed from the tibia and femurs with PBS through a 70 µm cell strainer. Cells were collected and suspended in RPMI 1640 medium (Gibco, USA) supplemented with 10% fetal bovine serum ( Gibco), 5% penicillin/streptomycin, 5% l‐glutamine plus 20 ng mL^−1^ GM‐CSF, and 10 ng mL^−1^ IL‐4.

##### Analysis of *α*‐GalCer‐diol Presentation by BMDCs

After 9 days of culturing, BMDCs were collected and seeded in 12‐well plates for glycolipid treatment for 24 h. BMDCs were then recollected and cocultured with splenocytes from corresponding glycolipid‐treated mice. After incubation for the designated time period, the intracellular level of IFN‐*γ* in CD4^+^ cells was determined by FACS. The supernatants were harvested to assay IL‐12/IL‐23/P40 concentration (NeoBioscience).

For the kinetics of *α*‐GalCer‐diol presentation, BMDCs were first seeded at a density of 5 × 10^5^cells per well in 1 mL culture medium in flat‐bottom 12‐well plates. After incubation with 300 × 10^−9^ m *α*‐GalCer‐diol or KRN7000 for different time periods, cells were collected and stained with PE‐conjugated antialpha‐GalCer:CD1d complex antibody (L363) (Thermo Fisher Scientific Inc., USA), allophycocyanin (APC)/Fire750‐conjugated antimouse CD11c antibody (N418), and fluorescein isothiocyanate (FITC)‐conjugated antimouse MHCII antibody (M5/114.15.2) (Biolegend Inc., San Diego, USA). The MFI of PE‐positive cells in gated CD11c^+^MHC II^+^ cells were analyzed using a FACS analyzer LSRII (BD Biosciences). To detect changes of the *α*‐GalCer‐CD1d complex, BMDCs were first pulsed with *α*‐GalCer‐diol or KRN7000 for 12 h, and were then washed and recultured at 37 °C. When harvested, cells were further stained with mAb L363 for FACS analysis. This experiment was also performed using DC2.4 cells.

##### Detection of Cell Subpopulations in the Spleen

Splenocytes were collected from glycolipid‐treated mice and diluted with staining solution (PBS (pH = 7.4) containing 0.1% bovine serum albumin). For further analysis of cell subpopulations, an array of antibodies was used for surface staining. All fluorescence‐conjugated antibodies were obtained from BioLegend as follows: FITC‐conjugated CD45 (30‐F11), PE‐conjugated antimouse CD54 (YN1/1.7.4), APC‐conjugated CD3 (145‐2C11), APC/Fire750‐conjugated CD8 (53–6.7), FITC‐conjugated CD4 (GK1.5), Brilliant Violet (BV) 421‐conjugated F4/80 (BM8123137), BV605‐conjugated CD11b (M1/70), APC/Fire750‐conjugated CD11c, PE‐conjugated NK1.1 (PK136), BV510‐conjugated Ly‐6C (HK1.4), and BV785‐conjugated Ly‐6G (1A8). After blocking with TruStain fcX (antimouse CD16/32) at 4 °C for 15 min, 2 × 10^6^ cells were stained with antibodies at 4 °C for 30 min, using 0.2 µg antibody per sample in 50 µL staining solution.

Analogously, isotypes were also applied in FACS analysis, including APC‐conjugated Armenian Hamster IgG, FITC‐conjugated rat IgG2b, APC/Fire750‐conjugated rat IgG2a, PE‐conjugated rat IgG1, PE‐conjugated rat Ig2b, PE‐conjugated mouse IgG2a, APC/Fire750‐conjugated Armenian Hamster IgG, FITC‐conjugated rat IgG2b, APC‐conjugated rat IgG2b, PE‐conjugated rat IgG2a, and PE/cy7‐conjugated rat IgG2b.k (Biolegend).

##### Detection of Intracellular IFN‐*γ*, IL‐4, and IL‐2

Splenocytes were first stained with cell surface markers, then washed, fixed, and permeabilized with BD Cytofix/Cytoperm solution according to the manufacturer's protocol. Fixed cells were then washed with BD Perm/Wash buffer and further stained with the indicated antibodies for cytokines. Peridinin‐chlorophyll‐protein complex‐cyanine 5.5 (Percp‐cy5.5)‐conjugated antimouse IFN‐*γ* Ab (XMG1.2), PE‐conjugated antimouse IL‐4 Ab (11B11), and BV 421‐conjugated antimouse Il‐2 Ab (JES6‐5H4) (BioLegend) were used for intracellular staining in different panels. BV 421‐conjugated rat IgG2b, PE‐conjugated rat IgG1, and Percp‐cy5.5‐conjugated Rat IgG1 (BioLegend) were used as isotype controls.

##### Analysis of Spleen iNKT Cell Activation

Splenocytes (2 × 10^6^/well in 12‐well plate) were stimulated with glycolipids at a concentration of 1.26 × 10^−9^ m for 16 h. Brefeldin A (eBioscience, Thermo Fisher), at 10 µg mL^−1^, was added for the last 2 h of incubation.^[^ ^36^ ^]^ After washing with cold PBS, cells were collected and incubated with 10 µL mCD1d/*α*‐GalCer Dextrame conjugated with R‐PE (PBS44 variant, YD8002‐PE, Immudex Fruebjergvej 3, Denmark) in 50 µL staining solution at room temperature for 20 min. The following antibodies (BioLegend) were further added to gate CD3^+^NK1.1^+^CD1d tetramer^+^ NKT cells: APC‐conjugated antimouse CD3 antibody and FITC‐conjugated antimouse NK1.1 antibody. Intracellular staining was performed using Percp‐cy5.5‐conjugated anti‐IFN‐*γ* mAb.

##### CD11b Neutralization and Monocyte/Macrophage Depletion

Mice were injected by I.P. injection with anti‐CSF‐1R (CD115) antibody (AFS98, Bio X Cell, West Lebanon, NH) or anti‐CD11b antibody (M1/70, Bio X Cell,) at a dose of 5 mg kg^−1^ body weight (in 300 µL dilution buffer, in vitro pure, pH = 7.4) 24 h before glycolipid administration. For the control group, the same amount of rat IgG2b (Bio X Cell) was administered. To characterize the efficacy of monocyte/macrophage depletion in vivo, mice were I.V. injected with clodronate liposomes (from Vrije University Amsterdam) at 5 mg kg^−1^ body weight in 200 µL PBS 48 h before glycolipid administration. For the control group, an equal amount of control PBS‐laden liposomes was administered. All liposomes were purchased from YEASEN Inc., Shanghai, China.

##### Examination of B16‐F10 Melanoma Lung Metastasis

To analyze the antitumor activity of glycolipids, the B16‐F10 melanoma lung metastasis model was established, as reported previously.^[^ ^56^ ^]^ Mice were administered 4 nmol (in 200 µL PBS) *α*‐GalCer‐diol or KRN7000 3 days prior to I.V. injection of 2 × 10^5^ B16‐F10 cells. Booster injections were performed 48 h later. The control group mice received vehicle solution only. Three weeks after tumor cell inoculation, mice were sacrificed and metastatic tumor nodules in the lung were examined.

##### Statistical Analysis

An independent *t*‐test was used to analyze the experimental data. All quantitative data were shown as the means ± standard error). Experimental data were analyzed by SPSS software. Statistical significance was defined as a *P* value of less than 0.05 (*) or 0.001 (#).

## Conflict of Interest

The authors declare no conflict of interest.

## Supporting information

Supporting Information
