# Supplementary material for: A Designed α‐GalCer Analog Promotes Considerable Th1 Cytokine Response by Activating the CD1d‐iNKT Axis and CD11b‐Positive Monocytes/Macrophages
Source: Adv Sci (Weinh). 2020 Jun 8;7(14):2000609. doi: 10.1002/advs.202000609 (PMC7375225; doi:10.1002/advs.202000609)
Supplement: Supplementary file 1 — Supporting Information [file ADVS-7-2000609-s001.pdf]

Designed α-GalCer Analog Promotes Considerable Th1 Cytokine Response through Activating the CD1d-iNKT Axis and CD11b-positive Monocytes/Macrophages

Juan Ma[#], *Peng He*[#], *Chuanfang Zhao*[#], *Quanzhong Ren, Zheng Dong, Jiahuang Qiu, Yang Jing, Sijin Liu, Yuguo Du*

Prof. J. Ma, Dr. P. He, Dr. C. Zhao, Mr. Q. Ren, Mr. Z. Dong, Dr. J. Qiu, Mr. Y. Jing, Prof. S. Liu, Prof. Y. Du

State Key Laboratory of Environmental Chemistry and Ecotoxicology

Research Center for Eco-Environmental Sciences

Chinese Academy of Sciences

Beijing 100085, P.R. China

Email: sjliu@rcees.ac.cn or duyuguo@rcees.ac.cn

Prof. J. Ma, Mr. Q. Ren, Mr. Z. Dong, Dr. J. Qiu, Prof. S. Liu

School of Environmental Sciences

University of Chinese Academy of Sciences

Beijing 100049, P.R. China

Dr. P. He, Dr. C. Zhao, Prof. Y. Du

School of Chemical Sciences

University of Chinese Academy of Sciences

Beijing 100049, P.R. China

Mr. Y. Jing

National Engineering Research Center for Carbohydrate Synthesis

Jiangxi Normal University

Nanchang 330022, Jiangxi, China

*: Correspondence to Sijin Liu, Ph.D. or Yuguo Du, Ph.D

#: These authors contributed equally to this work.

Table of Contents

Table S1. List of abbreviations..4

Table S2. Structure and activity of KRN7000 and some Th1-biased glycolipids...5

1.Supplementary Schemes...6

2.Supplementary Figures and figure legends 10

3.Reagents and instrumentation...16

4.Computational details...16

5.Synthestic procedures and characterization of intermediates and α-GalCer-diol 17

6.Purity analysis of α-GalCer-diol...35

7.Supporting information references 37

Table S1. List of abbreviations.

Abbreviation	Full name
**DCM**	dichloromethane
**PTSA**	p-toluenesulfonic acid
**LHMDS**	Lithium hexamethyldisilazide
**THF**	tetrahydrofuran
**EDCI**	1-ethyl-3(3-dimethylpropylamine) carbodiimide
**HOBt**	1-Hydroxybenzotriazole
**DMF**	N,N-Dimethylformamide
**TBDPSCl**	Tert-butylchlorodiphenylsilane
**BzCl**	Benzoyl chloride
**Pyr.**	pyridine
**TMSOTf**	Trimethylsilyl trifluoromethanesulfonate
**NIS**	N-iodosuccinimide
**TLC**	Thin Layer chromatography
**TMS**	tetramethylsilane
**ESI**	electrospray ionization
**MALDI-TOF**	matrix-assisted laser desorption/ionization time-of-flight
**PDB**	Protein Data Bank
**RMSD**	Root-mean-squaredeviation

Table S2. Structure and activity of KRN7000 and some Th1-biased glycolipids

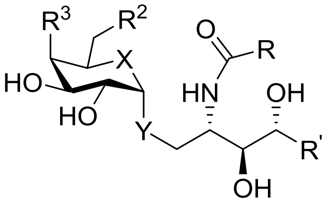

Glycolipids	X	Y	R	R′	R^2	R^3	Activity	Refs
KRN7000	O	O	$C_{25}H_{51}$	$C_{14}H_{29}$	OH	OH		
5″-C-α-GalCer	CH_2	O	$C_{25}H_{51}$	$C_{14}H_{29}$	OH	OH	IFN-γ (↑), IL-4↓	[1]
5″-S-α-GalCer	S	O	$C_{25}H_{51}$	$C_{14}H_{29}$	OH	OH	IFN-γ(—), IL-4↓	[2]
6″-Ar-α-GalCer	O	O	$C_{25}H_{51}$	$C_{14}H_{29}$	HNCONHAr	OH	IFN-γ(—), IL-4↓	[3]
4″-R-α-GalCer	O	O	$C_{25}H_{51}$	$C_{14}H_{29}$	OH	OR or OAr	IFN-γ(—), IL-4↓	[4-6]
α-C-GalCer	O	CH_2	$C_{25}H_{51}$	$C_{14}H_{29}$	OH	OH	IFN-γ(—), IL-4↓	[7, 8]
GC14_R	O	O	$(CH_2)_nAr$	$C_{14}H_{29}$	OH	OH	IFN-γ(—), IL-4↓	[9-11]
GC2Ph_25	O	O	$C_{25}H_{51}$	$(CH_2)_2Ph$	OH	OH	IFN-γ(—), IL-4↓	[10]

Collected data to look for the clues in designing new glycolipids. "↑"denotes increase , "↓"indicates reduction, and "—" means no significant change, relative to those in KRN7000.

1. Supplementary Scheme

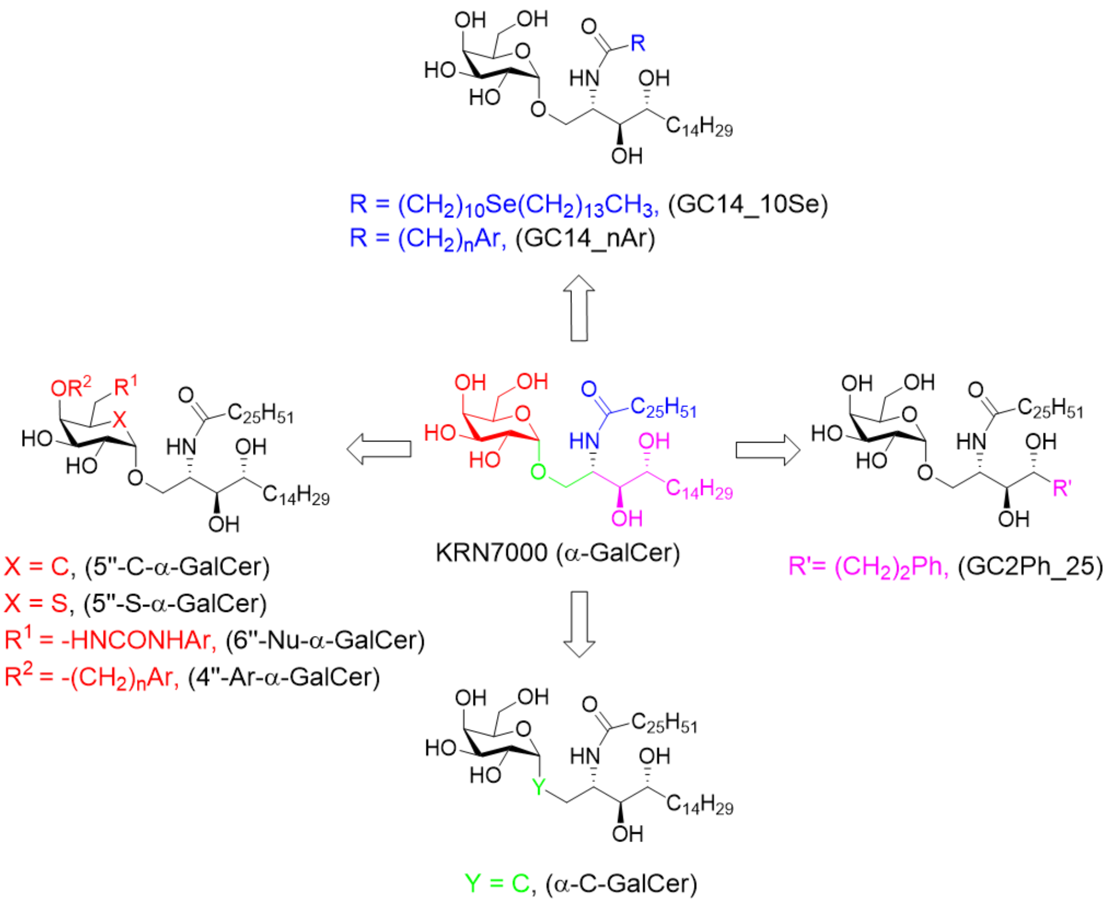

Scheme S1. The structures of KRN7000 and some reported Th1-biased glycolipids.

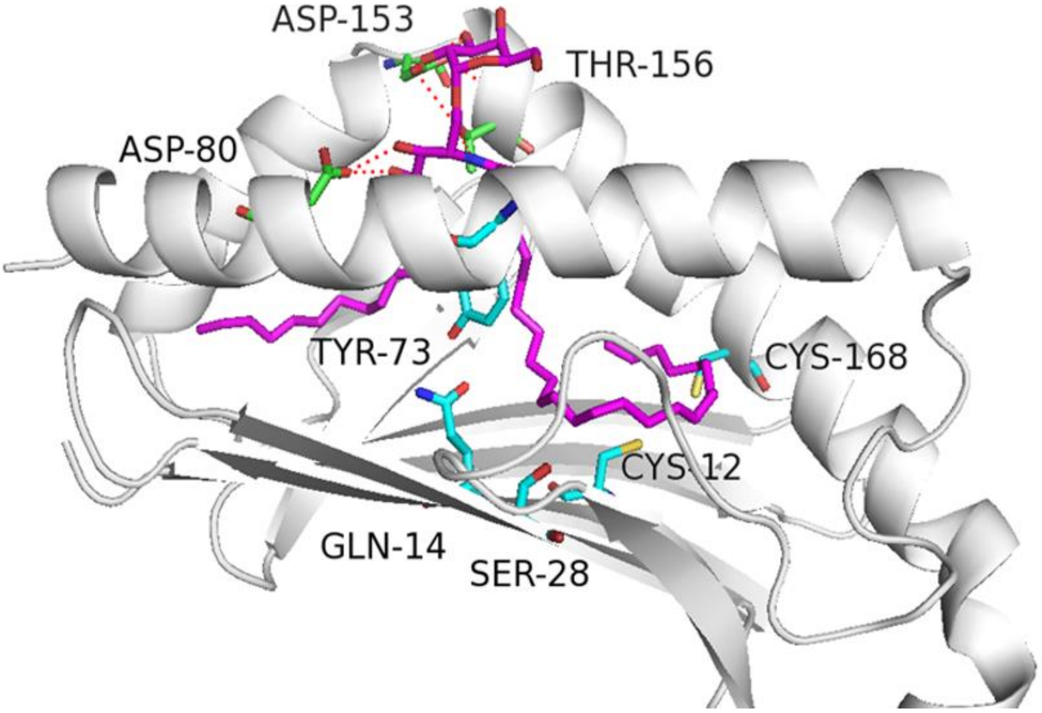

Scheme S2. Crystal structure of KRN7000-CD1d complex. For CD1d, carbon of the acyl chain, key residues and polar residues in the A pocket were colored by white, green and cyan, respectively. The KRN7000 was denoted by magenta and the hydrogen bonds formed between CD1d and KRN7000 were colored by a red dash.

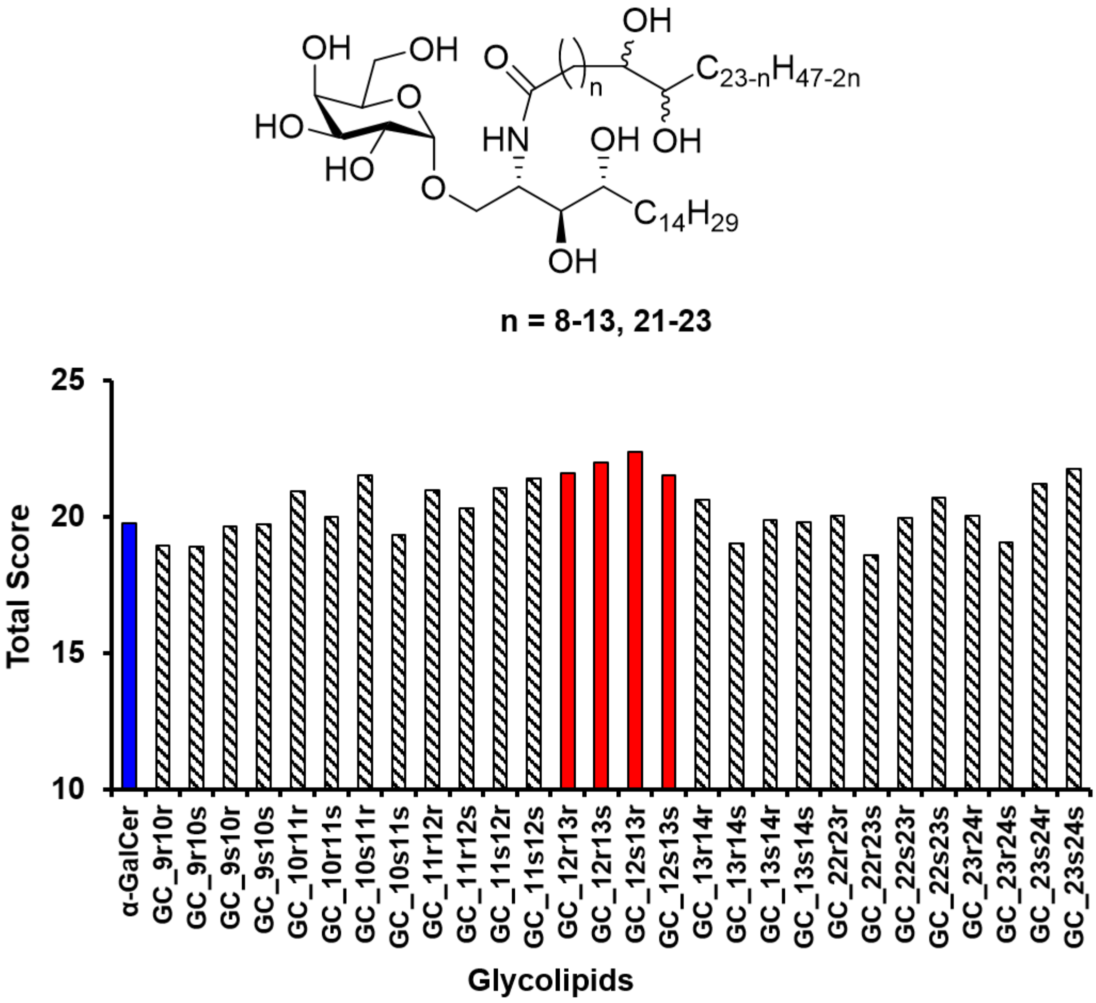

Scheme S3. Structures of glycolipids with vicinal diol on acyl chain and the TotalScore of them binding with CD1d. The histogram showing TotalScore of the docking between CD1d and glycolipid with diol at the 12th and 13th of acyl were colored by red.

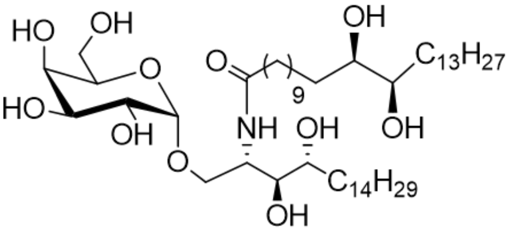

GC_12r13r

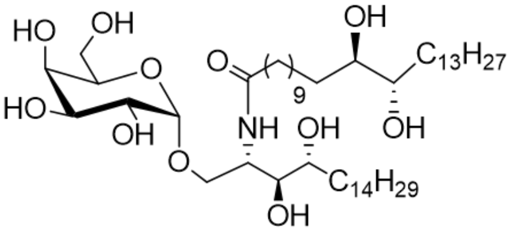

GC_12r13s

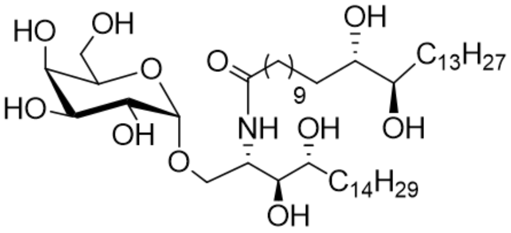

GC_12s13r

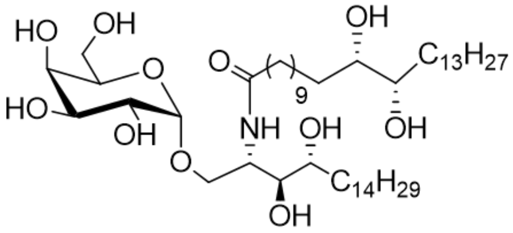

GC_12s13s

Scheme S4. The structures of 4 diastereomers of α-GalCer-diol.

2. Supplementary figures and figure legends

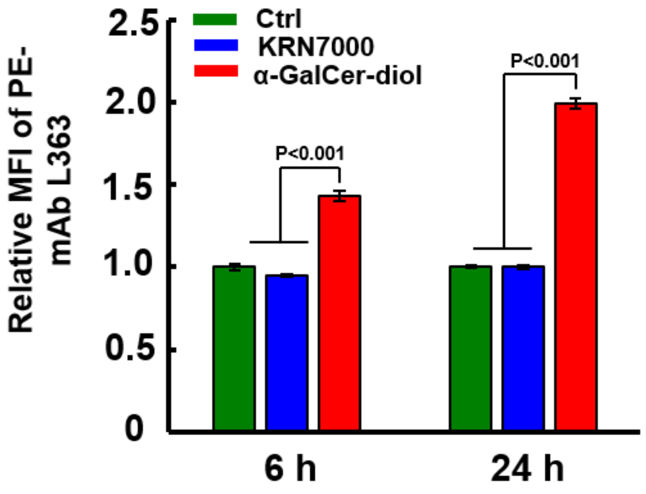

Figure S1. Enhanced interaction of α-GalCer-diol towards CD1d. Geo Mean quantification of L363 staining in DC2.4 cells following α-GalCer-diol or KRN7000 treatment for 6 hours and 24 hours relative to these in the control group was shown (n=5).

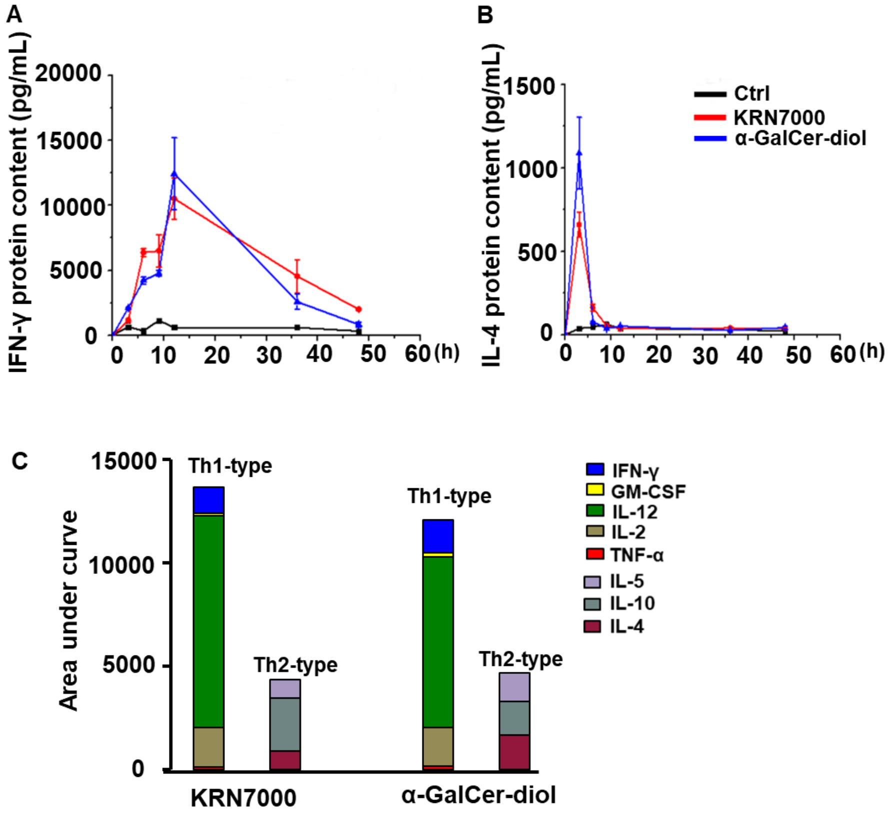

Figure S2. Assessment of Th1-type cytokine response induced by α-GalCer-diol. (A and B) IFN-γ and IL-4 in sera of mice that were I.V. administrated with 4 nmol α-GalCer-diol and KRN7000 over time (n=6). (A) Illustration of the AUC (area under the curve) of Th1 type cytokines (IFN-γ, IL-2, IL-12, TNF-α and GM-CSF) and Th2 type cytokines (IL-4, IL-5 and IL-10) in mice upon KNR7000 and α-GalCer-diol administration for 3, 6, 9, 12, 24 and 48 hours (n=4).

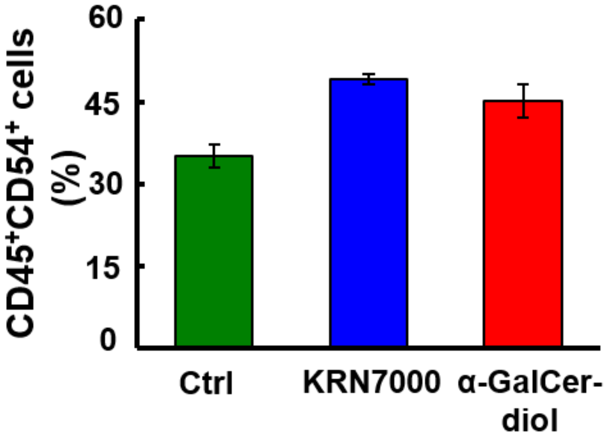

Figure S3. Remarkable inflammatory activation in the spleen. Following 4 nmol glycolipid exposure for 24 hours through I.V. administration, spleens were collected for further examination of $CD45^{+}CD54^{+}$ cells exclusion of red blood cell (n=4).

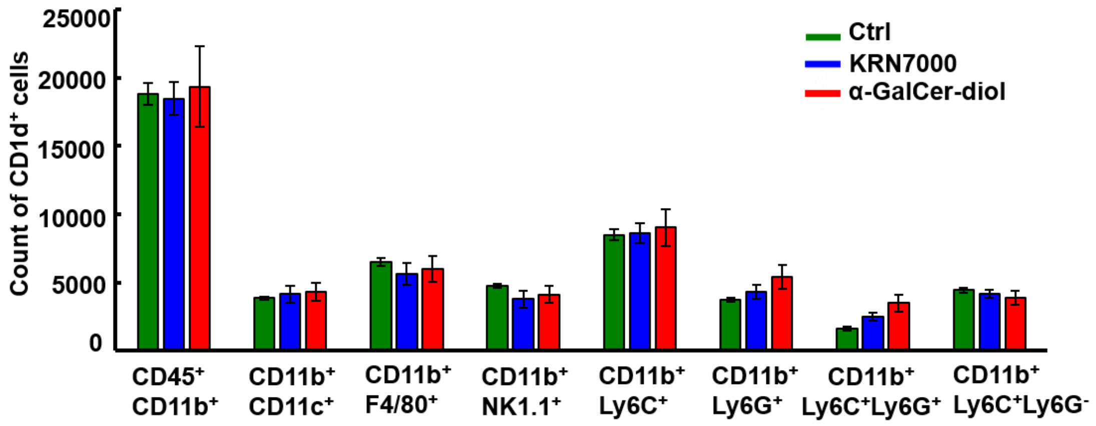

Figure S4. Assessment of CD1d proportion in subpopulations. Cell counting of PE-conjugated CD1d$^+$ proportions in each subpopulation of gated 500,000 splenocytes (n=5).

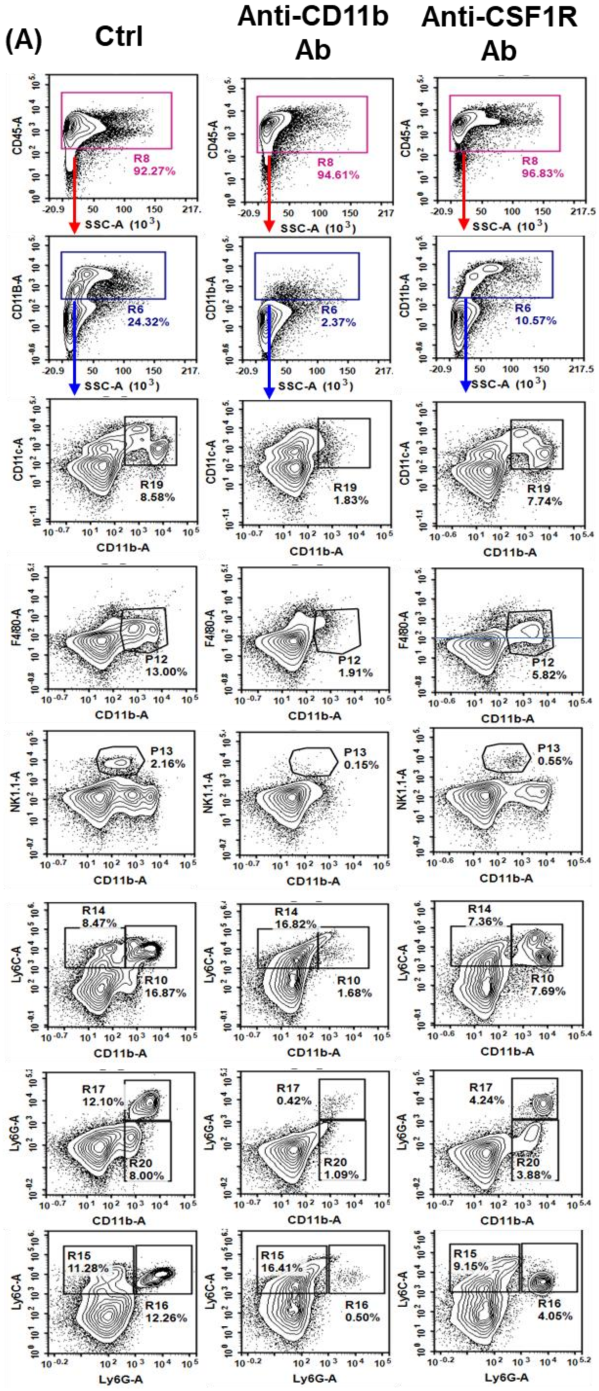

(B)

cell subtypes / (%)	Ctrl	Anti-CD11b Ab	Anti-CSF1R Ab
$CD45^{+}$	92.6±1.12	94.71±0.47	96.48±0.38
$CD11b^{+}$/ $CD45^{+}$	17.61±3.27	3.81±1.10#	11.96±0.99
CD11c+/ $CD45^{+}CD11b^{+}$	14.46±3.12	2.96±0.88#	8.38±0.34#
$F4/80^{+}$/ $CD45^{+}CD11b^{+}$	9.60±1.87	2.68±0.69#	6.60±0.40*
$NK1.1^{+}$/ $CD45^{+}CD11b^{+}$	1.42±0.25	0.36±0.10#	0.56±0.01#
$Ly6G^{+}$/ $CD45^{+}CD11b^{+}$	8.52±1.64	1.29±0.40#	4.24±0.05#
$Ly6C^{+}$/ $CD45^{+}CD11b^{+}$	12.24±2.50	2.74±0.84#	7.81±0.14#
$Ly6G^{-}Ly6C^{+}$/ $CD45^{+}CD11b^{+}$	10.25±1.83	13.24±1.45	10.13±0.54

Figure S5. Determination of the efficacy of neutralization and depletion. (A) Flow cytometry analysis of subpopulations gating in spleen cells from mice upon 5 mg/ kg body weight anti-CD11b antibody and anti-CSF1R antibody treatment for 24 hours. (B) Depletion analysis of each subpopulation upon antibody neutralization. Asterisk (*) indicates P<0.05, and the pound sign (#) denotes P<0.001, compared to the untreated control (n=5).

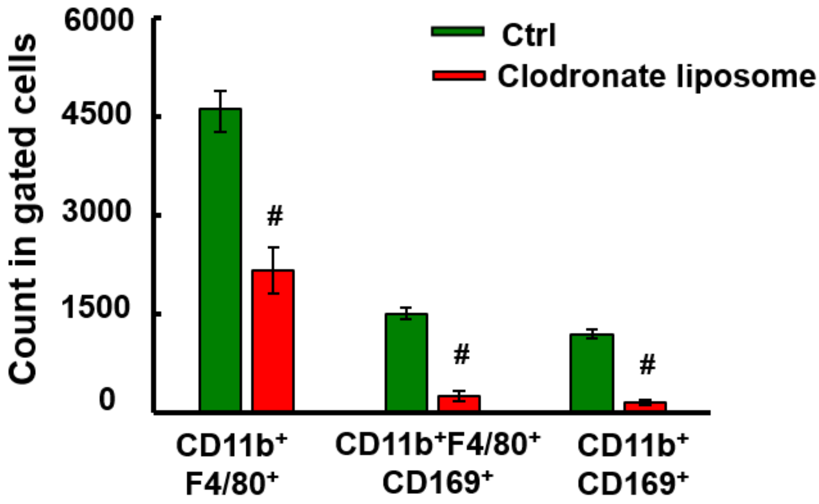

Figure S6. Analysis of the depletion effect upon clodronate liposome. Counting of $CD11b^{+}CD169^{+}$, $CD11b^{+}F4/80^{+}$ and $CD11b^{+}F4/80^{+}$ $CD169^{+}$ subpopulations in gated spleen cells from mice upon 5 mg/ kg body weight clodronate liposome administration for 48 hours. Pound sign (#) denotes P<0.001, compared to the control group (n=5).

3. Reagents and instrumentation

All chemicals used in this study were purchased from commercial suppliers. All moisture-sensitive reactions were carried out under a nitrogen atmosphere using flame-dried glassware. Anhydrous solvents were obtained following the standard procedures. ^{1}H NMR and ^{13}C NMR spectra were recorded with a Bruker AVANCE-III 400 MHz spectrometer (100 MHz for ^{13}C NMR). Chemical shifts were reported in ppm with respect to internal TMS. Mass spectra data were acquired with ESI on a Bruker micro-TOF Q II mass spectrometer or MALDI-TOF mass spectra on a Kratos MALDI II spectrometer. TLC was performed on silica gel HF_{254} through the detection by charring with 20% (v/v) H_2SO_4 in EtOH. Colum chromatography was performed on a column of silica gel (100-200 mesh). Solutions were concentrated in low temperature (below 50℃)

The target compound α-GalCer-diol and the reference compound KRN7000 were diluted in DMSO (*in vitro* pure, Solarbio Inc., Beijing, China), respectively, and warmed with water bath around 40℃ to ensure full dissolution prior to experimentation.

4. Computational details

Surflex-Dock was employed to mimic the interaction of designed α-GalCer-diol and CD1d with SYBYL 8.0 software (Tripos Inc., St. Louis, MO, USA). The crystal structure of CD1d was extracted from the PDB (PDB code is 3HE6;[12] www.rcsb.org). The A chain of 3HE6 was used for the study of the interaction between glycolipid and CD1d. The initial structure of KRN7000 was extracted from 3HE6, and the structures of designed glycolipids were derived from KRN7000. Water was removed from the PDB file before docking, and all the hydrogen atoms were added. Meanwhile, Gasteiger-Hückel atomic charge was added for ligands and proteins. To limit the effect of initial ligand conformation on docking result, 3 starting conformations of each ligand, obtained from ligand energy minimization with Max Iterations at 0, 100 or 1000 were

deliberately applied, respectively. Prior to docking, Protomol was generated with Threshold 0.5 and Bloat 10.0 Å. Parameters of Surflex-Dock were set at the default values, except the use of the extracted ligand of KRN7000 as a reference molecule. To check the feasibility of the parameters, KRN7000 was docked onto the binding site first. The RMSD of KRN7000 was 1.59 Å, indicating that the parameters used here were reasonable. The TotalScore of each ligand was used to judge their binding ability towards CD1d, followed by a computational structure analysis using PyMol software (https://pymol.org/2/).

5. Synthetic procedures and characterization of intermediates and α-GalCer-diol

6.1 Synthesis of compound 6

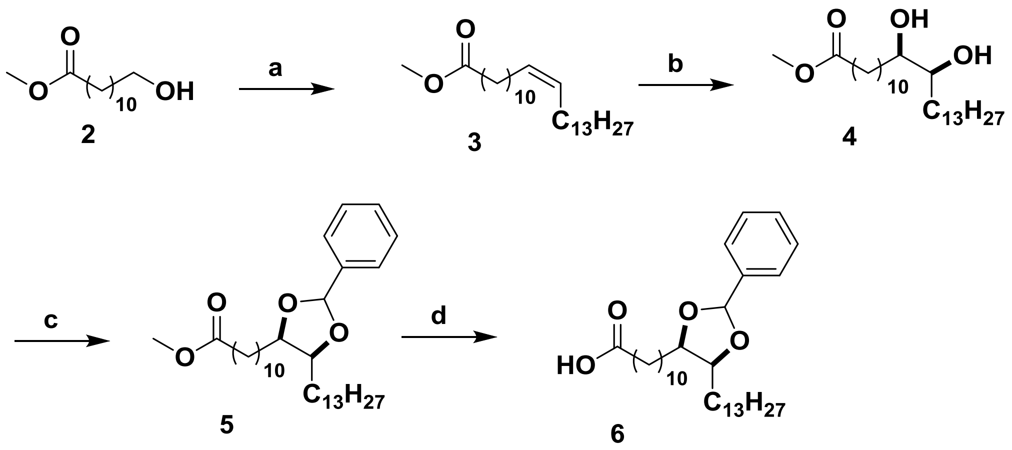

Scheme S5. Reagents and conditions: (a) (i) Dess-Martin periodinane, DCM, rt; (ii) $C_{14}H_{27}Ph_3P^+Br^-$, LHMDS, THF, -78°C to rt, 56% two steps; (b) AD-Mix-β, $CH_3SO_2NH_2$, tert-butyl alcohol/H_2O (1:1), 0°C, 78%; (c) Benzaldehyde dimethylacetal, PTSA, THF, 40°C, 92%; (d) LiOH·H_2O, THF/MeOH/H_2O (1:1:1); HCl aq., 94%.

6.2 Synthesis of α-GalCer-diol

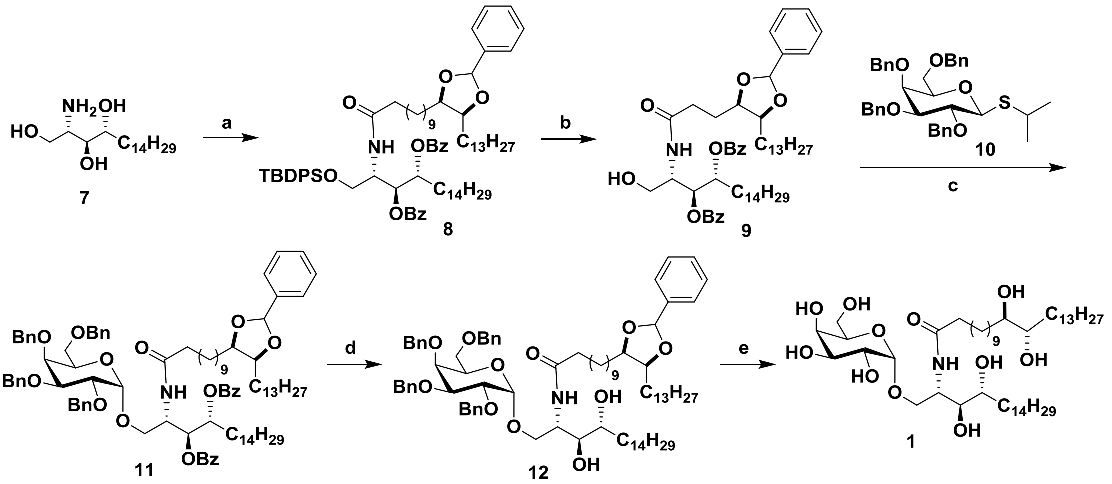

Scheme S6. Reagents and conditions: (a) (i) **6**, EDCI, HOBt, Et_3N, DMF; (ii) TBDPSCl, Pyr., rt; (iii) BzCl, 65% three steps; (b) HF-pyr., DCM, rt, 97%; (c) TMSOTf, NIS, DCM, molecular sieves 4 Å, N_2, 0°C, 64%; (d)NaOMe, MeOH, 98%; (e) 4 bar H_2, $Pd(OH)_2/C$, MeOH/EtOAc (4:1), 96%.

6.3 Synthetic procedures and characterizations

12-hexacosenoic acid methyl ester (3).

To a stirred solution of alcohol **2** (311 mg, 1.35 mmol) in CH_2Cl_2 (10 mL) at 0 °C was added Dess-Martin periodinane (746 mg, 1.76 mmol). The mixture was stirred at rt for 6 hours. The reaction was quenched by the addition of saturated aqueous $Na_2S_2O_3$ and saturated aqueous $NaHCO_3$ solution. The mixture was diluted with water and extracted with CH_2Cl_2 (3x30 mL), which was separated, dried (Na_2SO_4), and the solvent removed under reduced pressure. The resulting orange oil was used without purification. To a pre-cooled (-78 °C) solution of the salt $C_{14}H_{29}Ph_3P^+Br^-$ (800 mg, 1.48 mmol) in anhydrous THF was slowly added LHMDS (2.5M in THF, 0.59 ml, 1.48 mmol) under the protection of N_2. The mixture was stirred under these conditions for about 30 minutes, and then a solution of the pre-prepared aldehyde

(308 mg, 1.35 mmol) in dry THF (0.5mL) was added slowly to the reaction mixture at -78 °C via a syringe. The solution was stirred at this temperature for another 30 minutes, and then allowed to warm up to room temperature. The reaction was monitored by TLC (40:1 petroleum ether–EtOAc) until all starting materials disappeared. The reaction was then quenched by saturated NH_4Cl (0.3 mL), and the mixture was diluted with water and extracted with EtOAc (3x30 mL), which was separated, dried (Na_2SO_4), and the solvent removed under reduced pressure. The resulting orange oil was purified by column chromatography (silica gel, 2% EtOAc in Petroleum ether) to give **3** (309 mg, 56% for two steps, Z/E=5:1) as a colorless oil. ^1H NMR (400 MHz, $CDCl_3$) δ 5.41 – 5.29 (m, 2H), 3.66 (s, 3H), 2.30 (t, J = 7.6 Hz, 2H), 2.05 – 1.93 (m, 4H), 1.60 (td, J = 7.3, 4.6 Hz, 2H), 1.26 (m, 36H), 0.92 – 0.83 (m, 3H) ppm; ^{13}C NMR (100 MHz, $CDCl_3$) δ 174.5, 130.1, 130.0, 51.6, 51.6, 34.3, 32.1, 29.9, 29.9, 29.8, 29.7, 29.7, 29.6, 29.5, 29.5, 29.5, 29.4, 29.3, 27.4, 25.1, 22.9, 14.3 ppm; HRMS (ESI) m/z calcd for $C_{27}H_{52}O_2Na$ $[M+Na]^+$ 431.3860, found 431.3874.

^1H NMR (400 MHz, $CDCl_3$) spectrum of compound 3

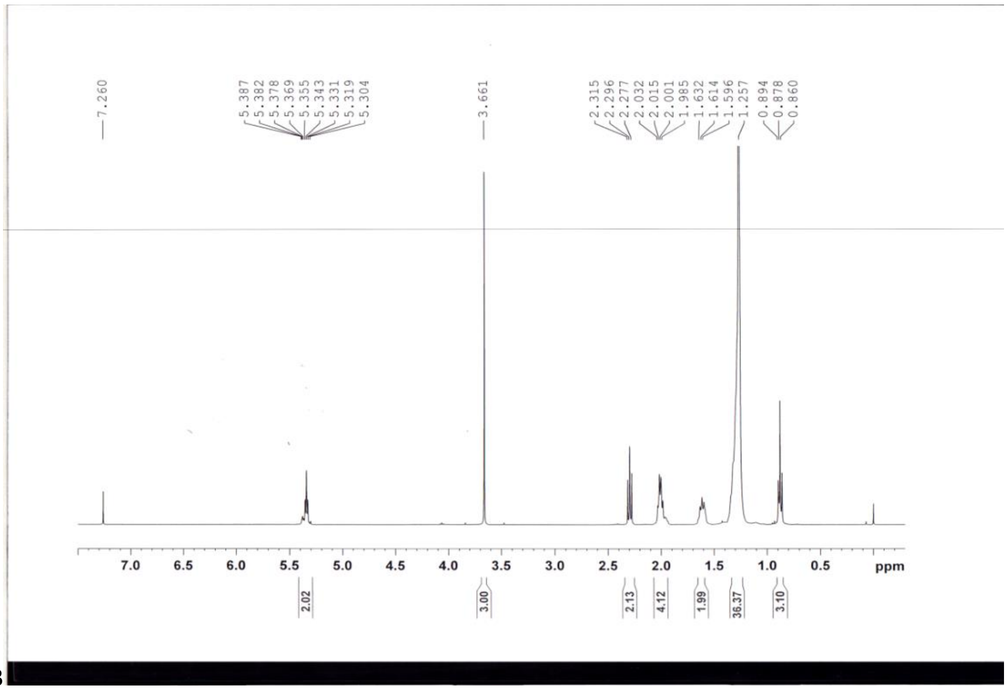

3

^{1}H NMR (400 MHz, $CDCl_3$) spectrum of compound 3

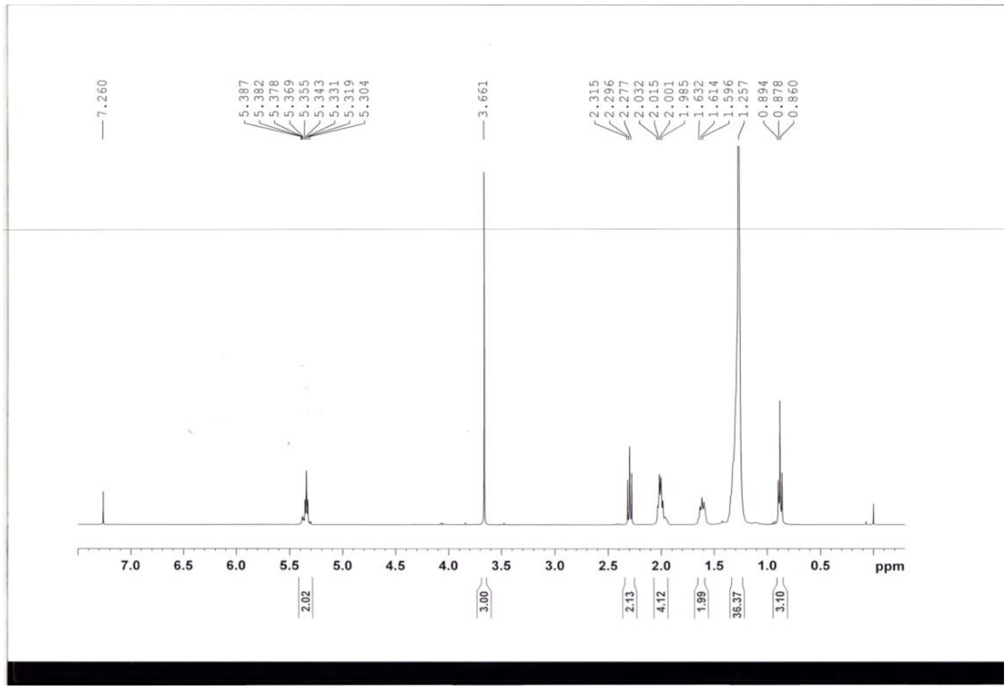

12, 13-dihydroxyhexacosenoic methyl ester (4)

To a pre-cooled (0 °C) solution of AD-Mix-β (2.81 g) in 40mL water and tert-butyl alcohol (V/V 1:1) was added methanesulfonamide (186 mg, 1.96 mmol). Olefin **3** (800 mg, 1.96 mmol) was then added, and the mixture was stirred at rt for 32 h. The reaction was quenched at 0 °C by the addition of sodium sulfite. Stirring was continued for an additional 45 minutes, and then the solution was extracted with CH_2Cl_2 (3 × 50 mL), which was separated, dried (Na_2SO_4), and the solvent removed under reduced pressure. The resulting solid was purified by column chromatography (silica gel, 50% EtOAc in Petroleum ether) to give **4** (676 mg, 78%) as a white solid: mp 111.2 °C; ^{1}H NMR (400 MHz, $CDCl_3$) δ 3.67 (s, 3H), 3.60 (t, J = 5.3 Hz, 2H), 2.30 (t, J = 7.5 Hz, 2H), 1.73 (d, J = 17.1 Hz, 3H), 1.61 (q, J = 7.3 Hz, 2H), 1.53 – 1.48 (m, 6H), 1.46 – 1.22 (m, 34H), 0.90 – 0.86 (m, 3H) ppm; ^{13}C NMR (100 MHz, $CDCl_3$) δ 174.5, 74.9, 74.8, 51.6, 34.3,

32.1, 31.4, 31.4, 29.8, 29.8, 29.8, 29.8, 29.8, 29.7, 29.6, 29.5, 29.5, 29.4, 29.3, 26.2, 26.2, 25.1, 22.8, 14.3 ppm; HRMS (ESI) m/z calcd for $C_{27}H_{54}O_4Na$ $[M+Na]^+$ 465.3914, found 465.3927.

^{1}H NMR (400 MHz, $CDCl_3$) spectrum of compound 4

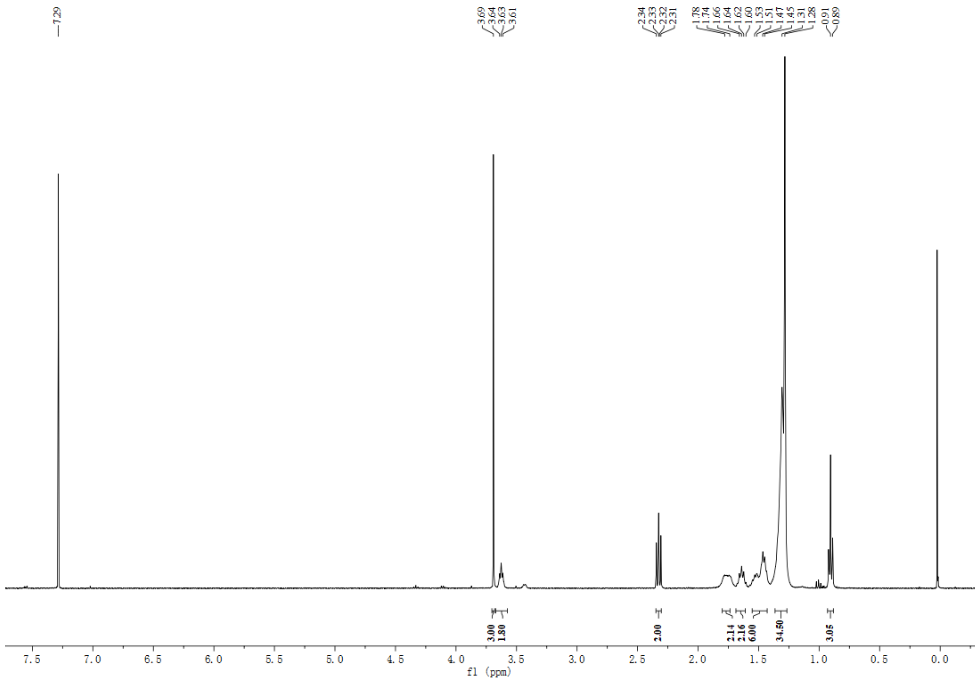

^{13}C NMR (100 MHz, $CDCl_3$) spectrum of compound 4

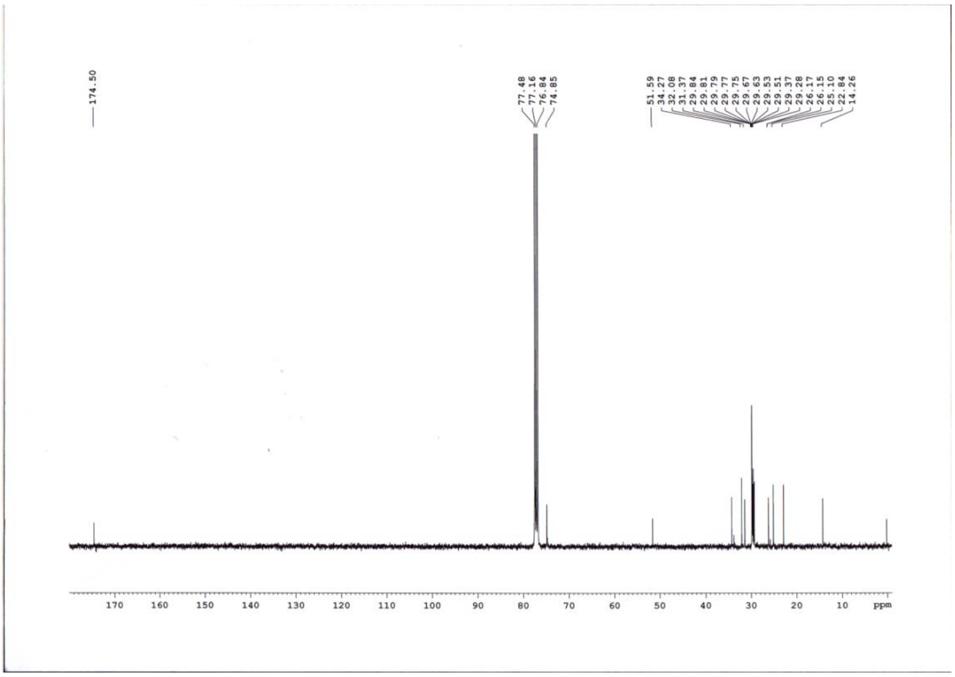

12, 13-benzylidenedihydroxyhexacosenoic methyl ester (5)

Benzaldehyde dimethylacetal (344 mg, 2.26 mmol) and p-toluenesulfonic acid (10 mg, 0.06 mmol) was added to **4** (500 mg, 1.13 mmol) in THF (20 mL). The reaction mixture was stirred at 40°C until the reaction was completed. A solution of $NaHCO_3$ was then added (3%, 50 mL). The resulting suspension was extracted with EtOAc (3× 50 mL), then the combined organic layers was washed with water (70 mL). The organic phase was dried over Na_2SO_4 and the solvent removed under reduced pressure. The resulting oil was purified by column chromatography (silica gel, 10% EtOAc in Petroleum ether) to give **5** (552 mg, 92%) as an oil. ^{1}H NMR (400 MHz, $CDCl_3$) δ 7.50 – 7.45 (m, 2H), 7.40 – 7.33 (m, 3H), 5.77 (s, 1H), 4.14 – 4.09 (m, 2H), 3.66 (s, 3H), 2.30 (t, J = 7.5 Hz, 2H), 1.66 – 1.53 (m, 6H), 1.52 – 1.44 (m, 2H), 1.42 – 1.22 (m, 34H), 0.92 – 0.83 (m, 3H) ppm; ^{13}C NMR (100 MHz, $CDCl_3$) δ 174.5, 138.3, 129.3, 128.4, 127.0, 103.2, 79.5, 79.4, 51.6, 34.3, 32.1, 30.1, 29.8, 29.8, 29.8, 29.8, 29.7, 29.7, 29.6, 29.5, 29.4, 29.3, 26.5, 25.1, 22.8, 14.3 ppm; HRMS (ESI) m/z calcd for $C_{34}H_{58}O_4Na$ $[M+Na]^+$ 553.4227, found 553.4239.

^{1}H NMR (400 MHz, $CDCl_3$) spectrum of compound 5

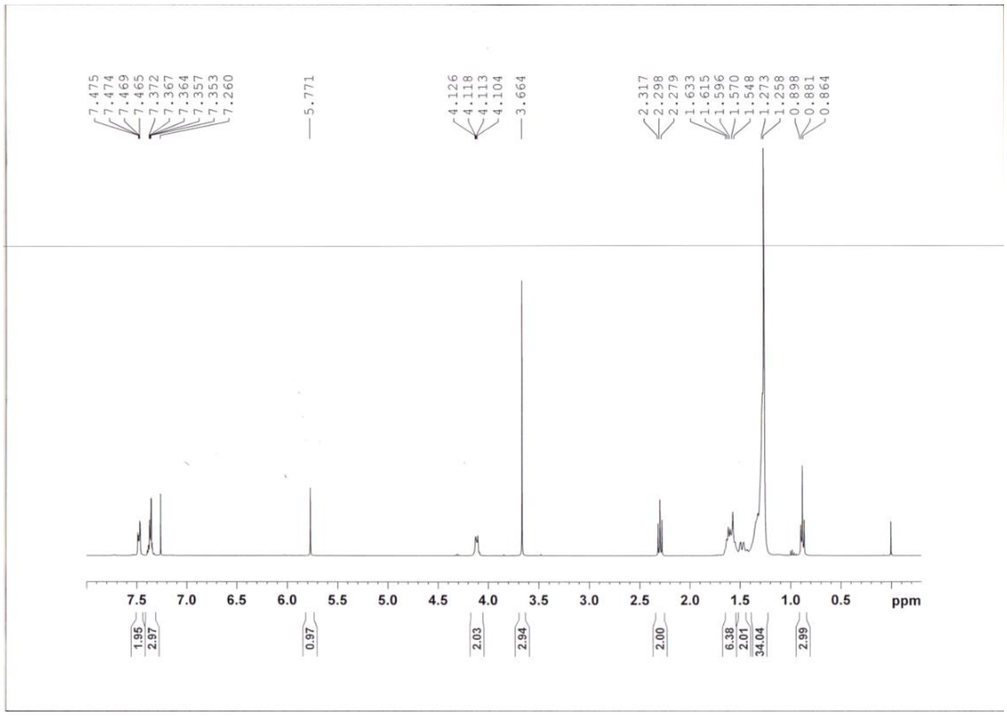

^{13}C NMR (100 MHz, $CDCl_3$) spectrum of compound 5

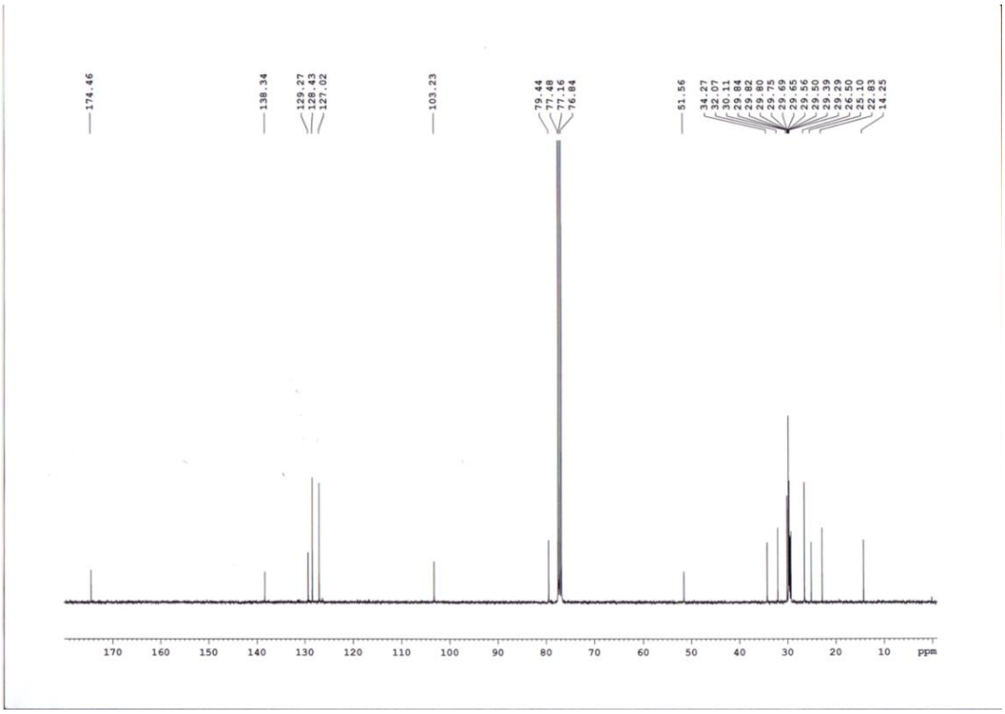

12, 13-benzylidenedihydroxyhexacosenoic acid (6)

To a solution of ester **5** (600 mg, 1.13 mmol) in THF, water and MeOH (V/V/V 1:1:1, 24 ml) was added LiOH·H_2O (95mg, 2.26 mmol). The mixture was stirred at rt overnight. The mixture was acidified to pH 3-4 (HCl. aq., 1N) 3-4 and extracted with DCM (3 x 30 ml). The combined organic extracts were washed with brine (40 ml), dried Na_2SO_4 and evaporated *in vacuo*. The resulting oil was purified by column chromatography (silica gel, 40% EtOAc in Petroleum ether) to give **6** (549 mg, 94%) as an oil. ^{1}H NMR (400 MHz, $CDCl_3$) δ 7.52 – 7.43 (m, 2H), 7.42 – 7.32 (m, 3H), 5.78 (s, 1H), 4.16 – 4.08 (m, 2H), 2.34 (t, J = 7.5 Hz, 2H), 1.66 – 1.53 (m, 6H), 1.52 – 1.44 (m, 2H), 1.39 – 1.22 (m, 34H), 0.92 – 0.84 (m, 3H) ppm; ^{13}C NMR (100 MHz, $CDCl_3$) δ 179.4, 138.3, 129.3, 128.4, 127.0, 103.2, 79.5, 79.4, 34.1, 32.1, 30.1, 29.8, 29.8, 29.8, 29.8, 29.8, 29.7, 29.6, 29.5, 29.5, 29.4, 29.2, 26.5, 24.8, 22.8, 14.3 ppm; HRMS (ESI) m/z calcd for $C_{33}H_{56}O_4Na$ $[M+Na]^+$ 539.4071, found 539.4089.

^{1}H NMR (400 MHz, $CDCl_3$) spectrum of compound 6

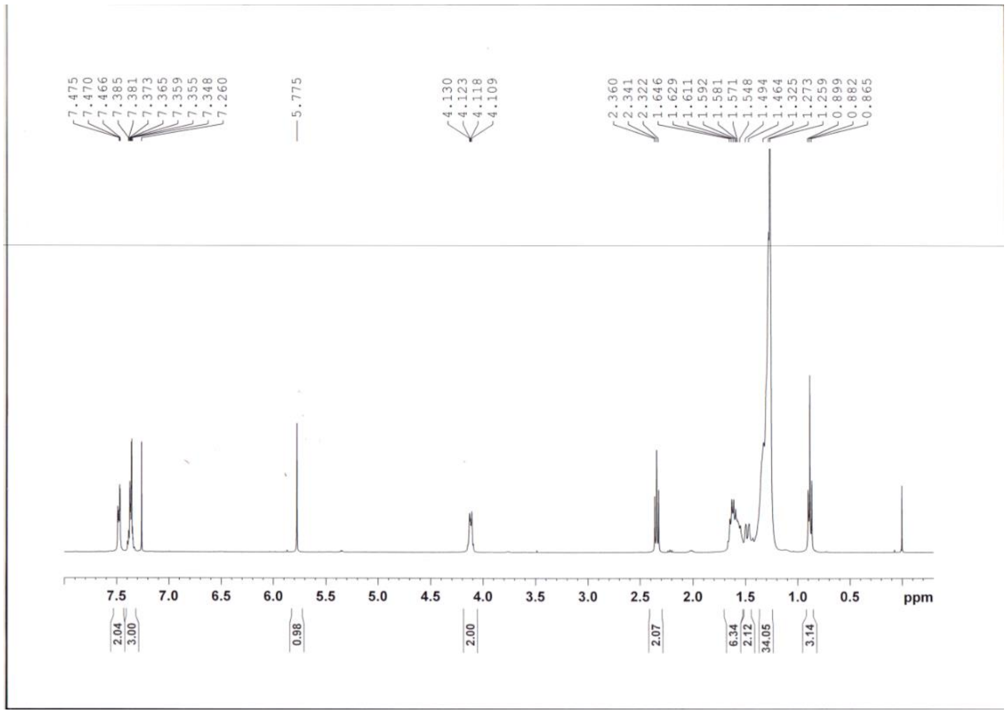

^{13}C NMR (100 MHz, $CDCl_3$) spectrum of compound 6

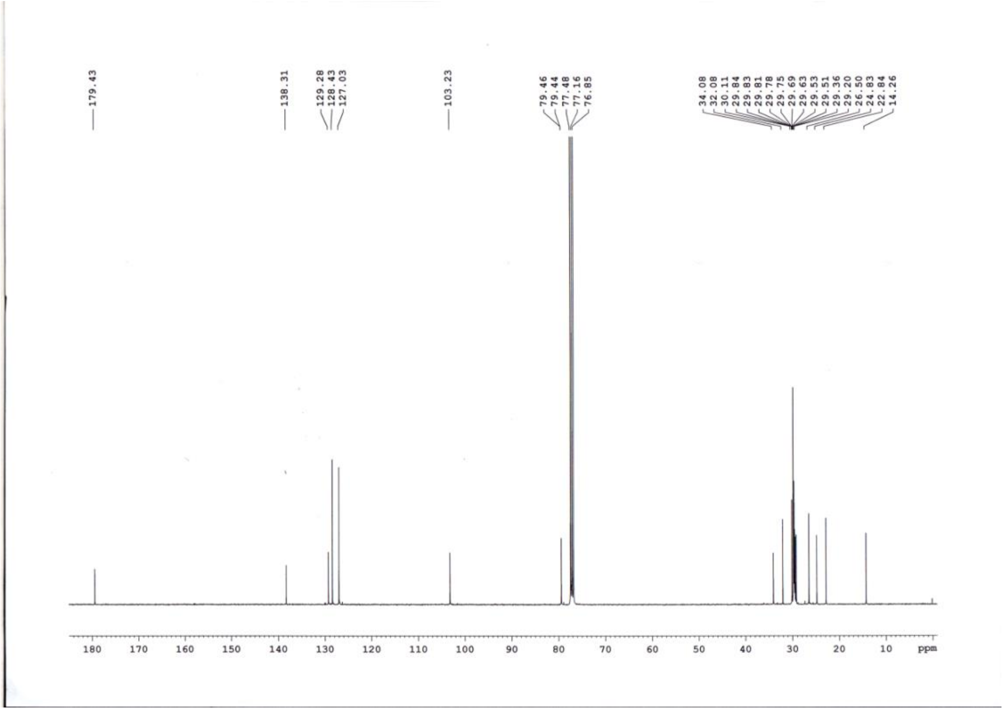

(2S,3S,4R)-2-N-(12,13-benzylidenedihydroxyhexacosenoylamino)-3,4-di-O-benzoyl- 1-O-(tert-Butyldiphenylsilyl)-1,3,4-octadecanetriol (8)

To a solution of acid **6** (800 mg, 1.55 mmol) in DMF (10.0 mL) was added HOBt (230 mg, 1.71 mmol) and EDCI (328 mg 1.71 mmol). The reaction mixture was stired at room temperature for 30 minutes, then phytosphingosine (492 mg, 1.55 mmol) and diisopropylethylamine (0.6 ml, 3.56 mmol) were added. After stirring at rt for 3h, and then the mixture was poured into 50 mL water. The resulting suspension was extracted with CH_2Cl_2 (3× 50 mL), then the combined organic layers were washed with water (70 mL). The organic phase was dried over Na_2SO_4 and the solvent removed under reduced to give crude amide as a white solid. The resulting crude amide was dissolved in pyridine (30 mL) and TBDPSCl (0.48 mL, 1.86 mmol) was added. After TLC analysis showed full consumption of the starting material (18 hours), BzCl (0.71ml, 6.20 mmol) was added and the mixture was kept at rt for 12 hours, 4 mL MeOH was

added and the mixture was stirred at room temperature for 1hours. The solvent was evaporated *in vacuo* and the resulting oil was taken up in ethyl acetate and washed with brine, then the organic layer was dried (Na_2SO_4), filtered and concentrated. The resulting oil was purified by column chromatography (silica gel, 10% EtOAc in Petroleum ether) to give **8** (1.29g, 65% for three steps) as an oil. ^{1}H NMR (400 MHz, $CDCl_3$) δ 7.97 (ddd, J = 7.2, 6.2, 1.4 Hz, 4H), 7.63 – 7.55 (m, 3H), 7.55 – 7.50 (m, 1H), 7.49 – 7.42 (m, 6H), 7.41 – 7.29 (m, 8H), 7.28 – 7.23 (m, 2H), 7.10 (t, J = 7.5 Hz, 2H), 6.04 (d, J = 9.4 Hz, 1H), 5.76 (d, J = 11.9 Hz, 2H), 5.34 (dt, J = 10.0, 2.9 Hz, 1H), 4.50 (td, J = 9.2, 4.7 Hz, 1H), 4.18 – 4.08 (m, 2H), 3.80 (dd, J = 10.7, 3.1 Hz, 1H), 3.70 (dd, J = 10.6, 3.0 Hz, 1H), 2.18 (td, J = 7.4, 4.3 Hz, 2H), 1.97 – 1.75 (m, 2H), 1.66 – 1.53 (m, 6H), 1.52 – 1.44 (m, 2H), 1.41 – 1.16 (m, 58H), 0.99 (s, 9H), 0.87 (td, J = 6.9, 2.4 Hz, 6H) ppm; ^{13}C NMR (100 MHz, $CDCl_3$) δ 172.9, 166.5, 165.2, 143.4, 138.3, 133.2, 133.0, 130.3, 130.0, 129.9, 129.9, 129.3, 128.7, 128.5, 128.5, 128.4, 127.9, 127.1, 127.0, 103.2, 86.9, 79.4, 79.4, 74.3, 72.9, 61.8, 48.7, 37.0, 32.1, 30.1, 30.1, 29.8, 29.8, 29.8, 29.8, 29.7, 29.7, 29.7, 29.7, 29.6, 29.5, 29.5, 28.6, 26.6, 26.5, 25.9, 25.8, 22.8, 14.3 ppm; MALDI-TOF MS m/z calcd for $C_{81}H_{119}NO_8SiNa$ $[M+Na]^+$ 1284.9, found 1284.9.

^{1}H NMR (400 MHz, $CDCl_3$) spectrum of compound 8

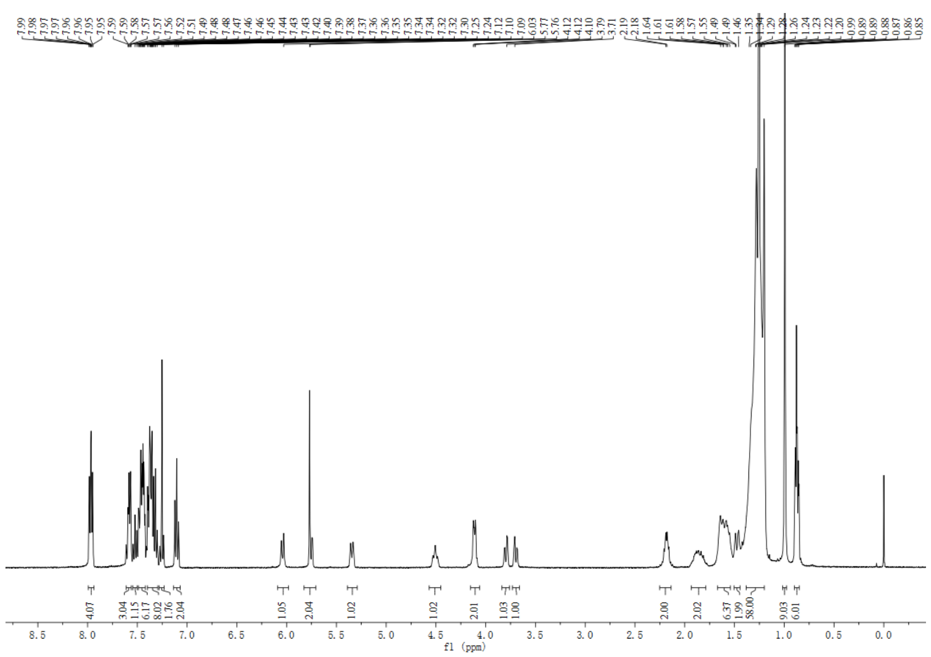

^{13}C NMR (100 MHz, $CDCl_3$) spectrum of compound 8

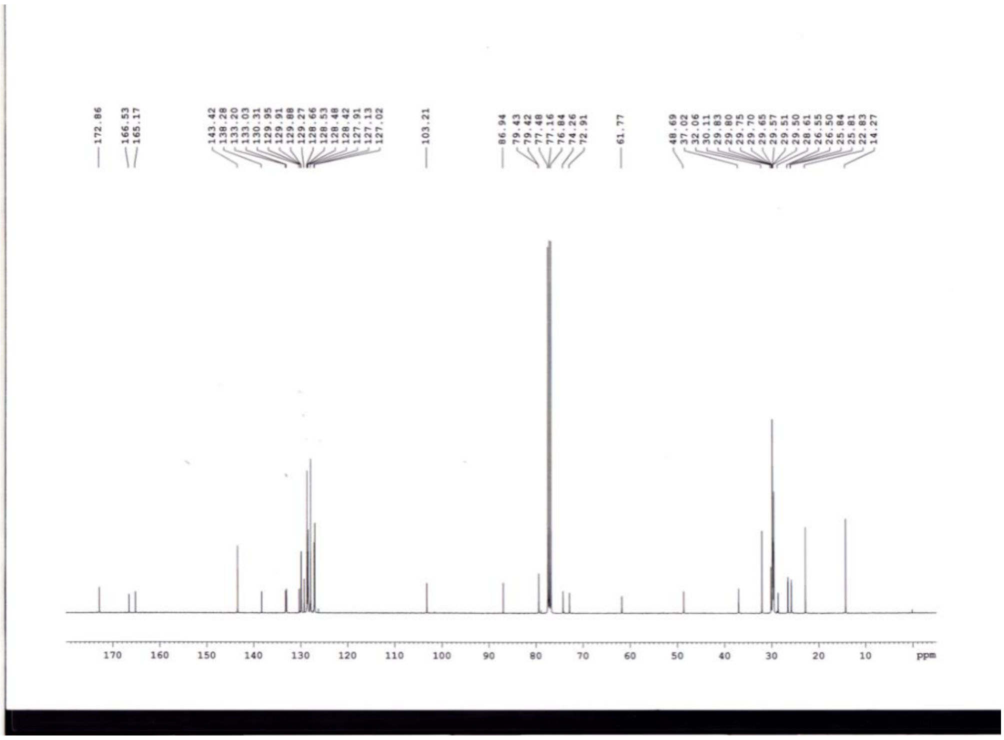

(2S,3S,4R)-2-N-(12,13-benzylidenedihydroxyhexacosenoylamino)-3,4-di-O-benzoyl- 1,3,4-octadecanetriol (9)

Compound **8** (500 mg, 0.40 mmol) was dissolved in DCM (10 mL). The mixture was cooled to 0 °C and 70% hydrogen fluoride-pyridine complex was added (150 µL, 1.22 mmol). The reaction stirred at rt for 5h. Following this, the mixture was washed with aqueous $NaHCO_3$ solution (2×5 mL), brine (1×5 mL), and dried over Na_2SO_4. The solvent was evaporated *in vacuo* and purified by silica gel chromatography (40% EtOAc in Petroleum ether) to yield **9** as a colourless oil (395 mg, 97%). ^{1}H NMR (400 MHz, $CDCl_3$) δ 8.09 – 8.02 (m, 2H), 7.97 – 7.92 (m, 2H), 7.67 – 7.60 (m, 1H), 7.49 (tt, J = 9.3, 7.2 Hz, 5H), 7.40 – 7.32 (m, 5H), 6.39 (d, J = 9.2 Hz, 1H), 6.03 (s, 0H), 5.77 (s, 1H), 5.42 – 5.33 (m, 2H), 4.39 (tt, J = 9.3, 2.6 Hz, 1H), 4.20 – 4.07 (m, 2H), 3.64 (qd, J = 12.3, 2.6 Hz, 2H), 2.83 (s, 1H), 2.36 – 2.23 (m, 2H), 2.02 (q, J = 8.6,

7.4 Hz, 2H), 1.73 – 1.52 (m, 6H), 1.51 – 1.42 (m, 2H), 1.40 – 1.20 (m, 58H), 0.88 (t, J = 6.6 Hz, 6H) ppm; ^{13}C NMR (100 MHz, $CDCl_3$) δ 173.4, 167.3, 166.4, 138.3, 134.0, 133.2, 130.1, 130.0, 129.8, 129.3, 129.2, 128.8, 128.8, 128.5, 128.4, 127.0, 126.3, 103.2, 101.6, 79.4, 79.4, 78.9, 77.4, 74.1, 73.9, 61.7, 50.1, 37.0, 32.1, 30.1, 30.1, 29.9, 29.8, 29.8, 29.8, 29.8, 29.7, 29.7, 29.6, 29.5, 29.5, 29.5, 29.5, 28.6, 26.5, 26.5, 26.0, 25.9, 22.8, 14.3 ppm; MALDI-TOF MS m/z calcd for $C_{65}H_{101}NO_8Na$ $[M+Na]^+$ 1046.7, found 1046.7.

^{1}H NMR (400 MHz, $CDCl_3$) spectrum of compound 9

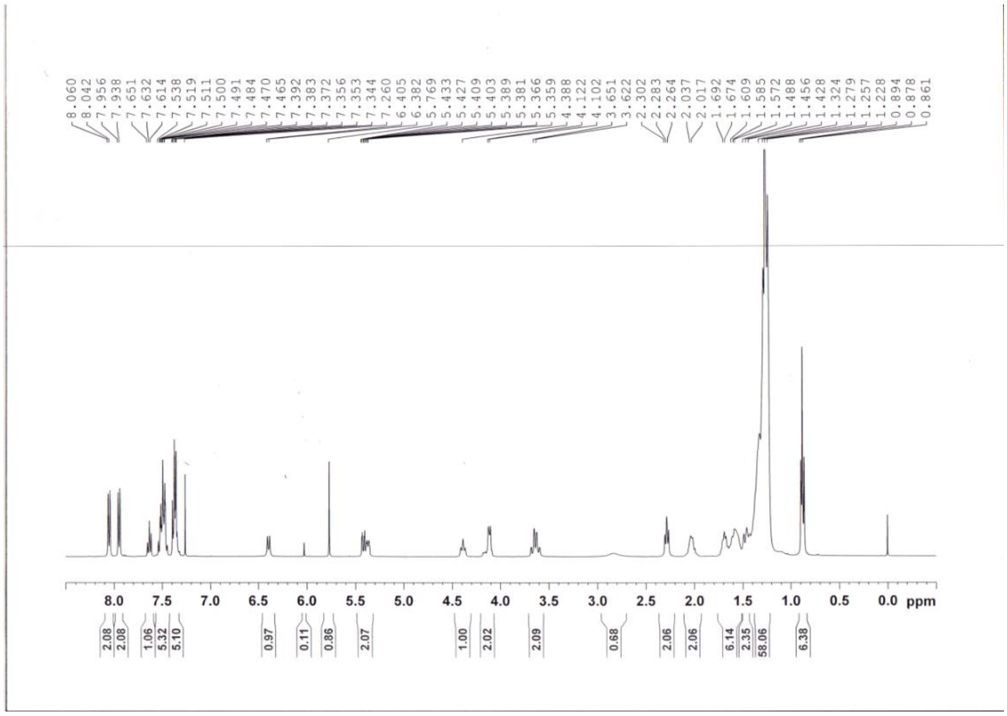

^{13}C NMR (100 MHz, $CDCl_3$) spectrum of compound 9

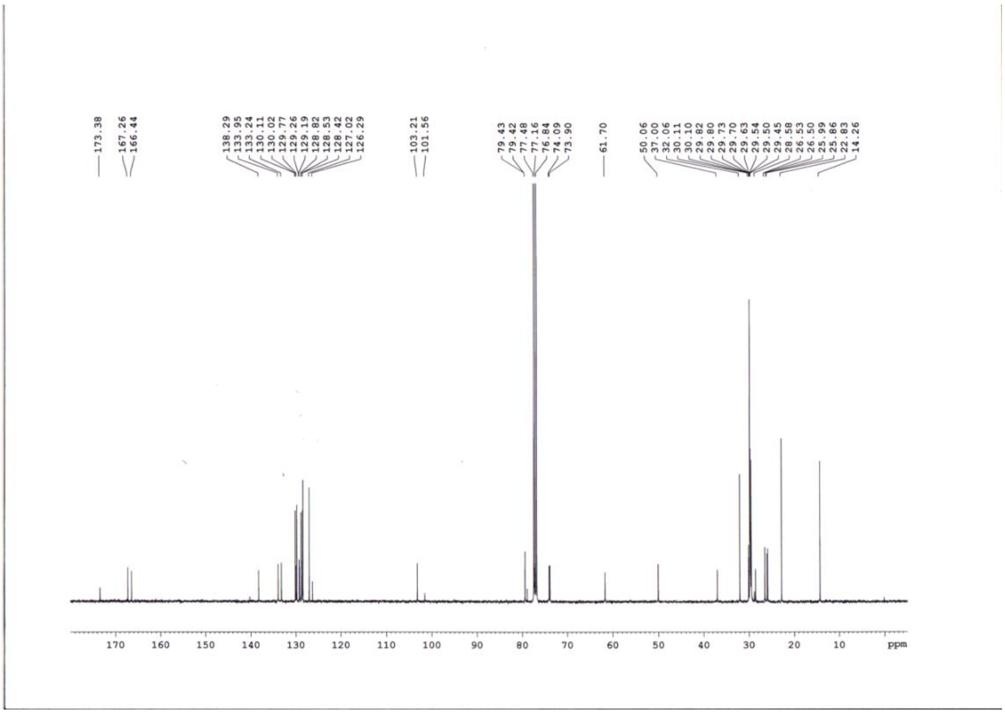

(2S,3S,4R)-2-N-(12,13-benzylidenedihydroxyhexacosenoylamino)-3,4-di-O-benzoyl-1-O-(2,3,4,6-tetra-O-benzyl-α-D-galactopyranosyl)-1,3,4-octadecanetriol (11)

A solution of **9** (208 mg, 0.20 mmol) and **10** (129 mg, 0.22 mmol) in dichloromethane (5 ml) was stirred in the presence of 4 Å molecular sieves (50mg, powdered) for 15 minutes. The mixture was cooled (0°C) and then NIS (50 mg, 0.22 mmol) and TMSOTf (2 µL, 11 µmol) were added. The mixture was kept at 0°C for 5h. The reaction mixture was quenched with triethylamine, and aqueous sodium thiosulfate (15%, 20 mL) was added to the mixture. The mixture was washed with brine (2×15 mL). The organic phase was dried (Na_2SO_4), filtered, and the filtrate was concentrated *in vacuo*. The resulting oil was purified by silica gel chromatography (15% EtOAc in Petroleum ether) to yield **11** as a colourless oil (192 mg, 64%). ^{1}H NMR (400 MHz, $CDCl_3$) δ 8.07 – 7.99 (m, 2H), 7.95 – 7.88 (m, 2H), 7.63 – 7.56 (m, 1H), 7.48 (dq, J = 17.0, 7.5 Hz, 5H), 7.39 – 7.31 (m, 9H), 7.31 – 7.17 (m, 20H), 6.87 (d, J = 9.6 Hz, 1H),

5.77 (s, 1H), 5.67 (dd, J = 9.8, 2.8 Hz, 1H), 5.41 (dt, J = 7.5, 3.4 Hz, 1H), 4.86 (d, J = 11.5 Hz, 1H), 4.76 – 4.70 (m, 2H), 4.64 (dd, J = 12.1, 6.0 Hz, 3H), 4.59 – 4.49 (m, 3H), 4.41 (d, J = 12.1 Hz, 1H), 4.19 – 4.07 (m, 3H), 4.08 – 3.97 (m, 2H), 3.90 – 3.78 (m, 2H), 3.60 (dd, J = 12.0, 2.6 Hz, 1H), 3.51 (dd, J = 9.5, 7.2 Hz, 1H), 3.29 (dd, J = 9.5, 5.4 Hz, 1H), 2.14 (t, J = 8.1 Hz, 2H), 1.88 (d, J = 6.2 Hz, 2H), 1.67 – 1.53 (m, 6H), 1.43 – 1.14 (m, 60H), 0.87 (td, J = 6.9, 2.2 Hz, 6H) ppm; ^{13}C NMR (100 MHz, $CDCl_3$) δ 173.3, 166.1, 165.3, 138.9, 138.5, 138.5, 138.3, 137.8, 133.4, 133.0, 130.3, 130.1, 129.9, 129.9, 129.3, 128.8, 128.7, 128.5, 128.5, 128.4, 128.3, 128.3, 128.3, 128.1, 128.1, 127.9, 127.8, 127.8, 127.7, 127.6, 127.0, 126.3, 103.2, 101.6, 100.6, 79.4, 79.4, 78.9, 78.8, 77.4, 76.7, 75.0, 74.8, 73.9, 73.6, 73.4, 73.4, 72.5, 70.9, 70.5, 69.3, 48.8, 36.8, 32.1, 30.1, 30.1, 29.9, 29.9, 29.8, 29.8, 29.8, 29.8, 29.8, 29.7, 29.7, 29.6, 29.6, 29.5, 29.5, 28.9, 28.7, 26.6, 26.5, 25.8, 25.8, 22.8, 14.3 ppm; MALDI-TOF MS m/z calcd for $C_{99}H_{135}NO_{13}Na$ $[M+Na]^+$ 1569.0, found 1569.1.

^{1}H NMR (400 MHz, $CDCl_3$) spectrum of compound 11

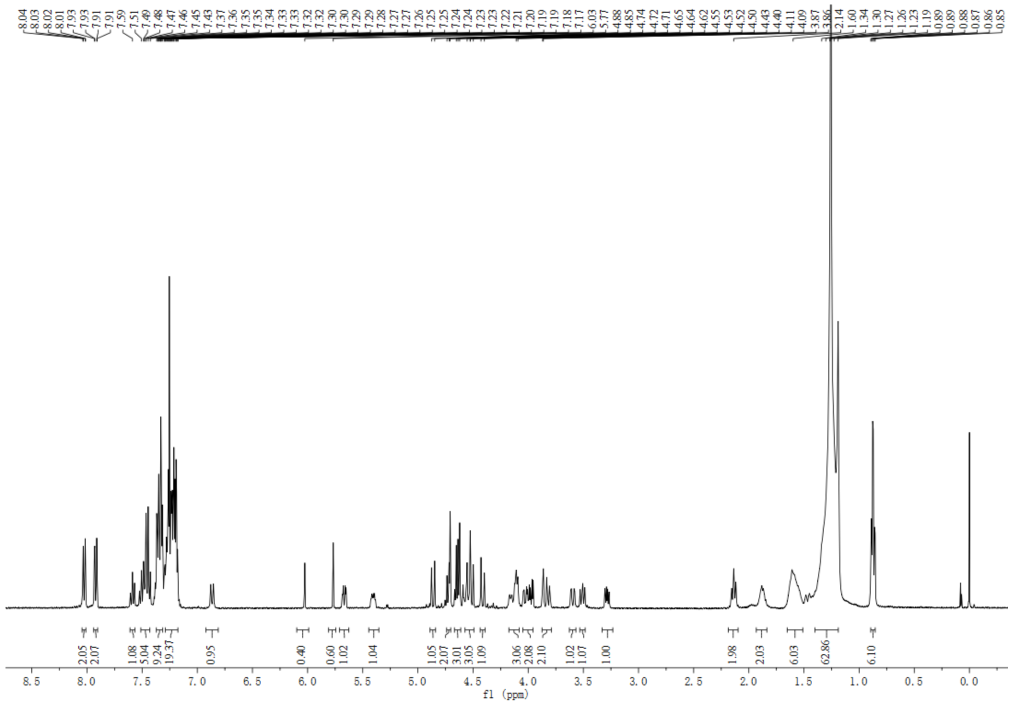

^{13}**C NMR (100 MHz, CDCl$_3$) spectrum of compound 11**

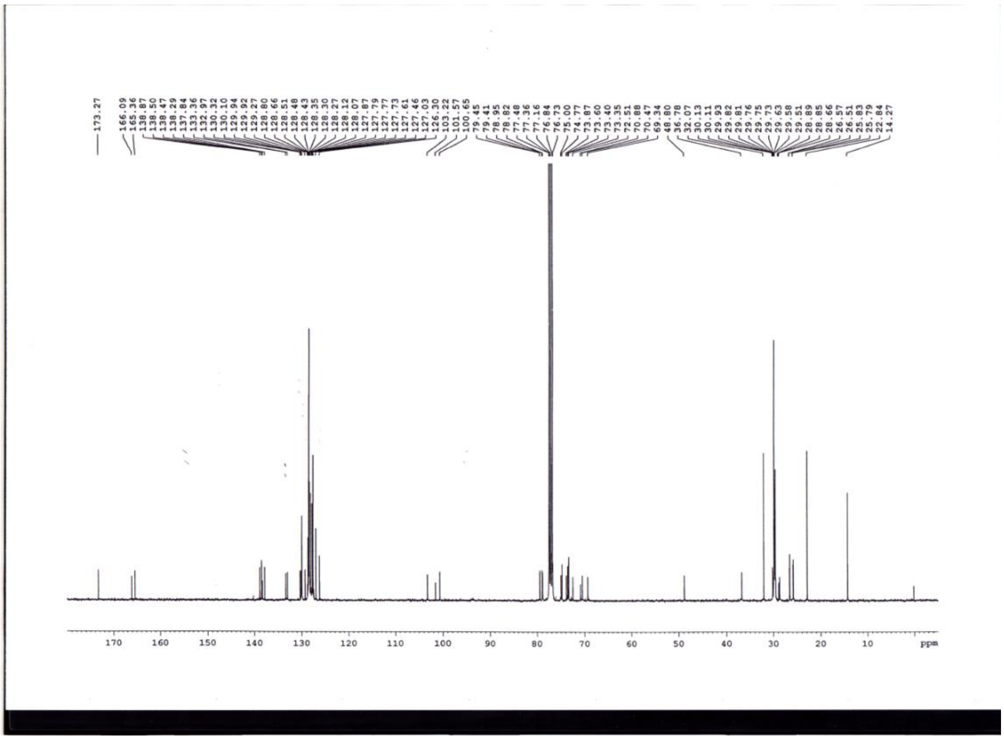

(2S,3S,4R)-2-N-(12,13-benzylidenedihydroxyhexacosenoylamino)-3,4-dihydroxyl-1-O-(2,3,4,6-tetra-O-benzyl-α-D-galactopyranosyl)-1,3,4-octadecanetriol (12)

To a solution of **11** (148 mg, 0.096 mmol) in anhydrous methanol (4 mL) was added a 1M solution of sodium methoxide in MeOH until pH 9-10 was reached. The reaction mixture was stirred at rt until the reaction was completed. The reaction was neutralized with amberlite IR-120 (H+), dissolved with CH_2Cl_2, and filtered. the filtrate was concentrated *in vacuo* and purified by silica gel chromatography (40% EtOAc in Petroleum ether) to yield **12** as a colourless oil (126 mg, 98%). ^{1}H NMR (400 MHz, CDCl$_3$) δ 7.55 – 7.45 (m, 2H), 7.44 – 7.24 (m, 23H), 6.41 (d, J = 8.4 Hz, 1H), 5.78 (s, 1H), 4.99 – 4.84 (m, 3H), 4.79 (d, J = 2.5 Hz, 2H), 4.71 (d, J = 11.6 Hz, 1H), 4.59 (d, J = 11.4 Hz, 1H), 4.50 (d, J = 11.6 Hz, 1H), 4.41 (d, J = 11.6 Hz, 1H), 4.28 – 4.12 (m, 2H), 4.07 (dd, J = 10.0, 3.7 Hz, 1H), 4.00 (d, J = 2.7 Hz, 1H), 3.96 – 3.86 (m, 4H), 3.53 – 3.41 (m, 4H), 2.14 (t, J = 7.6 Hz, 2H), 1.72 – 1.45 (m, 6H), 1.42 – 1.21 (m, 62H), 0.91

(t, J = 6.7 Hz, 6H) ppm; ^{13}C NMR (100 MHz, $CDCl_3$) δ 173.2, 138.5, 138.5, 138.0, 137.7, 129.3, 128.8, 128.6, 128.6, 128.6, 128.5, 128.4, 128.4, 128.3, 128.3, 128.1, 128.0, 127.8, 127.8, 127.6, 127.0, 126.3, 103.2, 101.6, 99.4, 79.5, 79.4, 78.9, 77.4, 76.4, 76.2, 74.9, 74.6, 74.4, 73.8, 73.4, 72.9, 70.2, 70.1, 69.1, 49.7, 36.9, 33.4, 32.1, 30.1, 29.9, 29.8, 29.8, 29.7, 29.7, 29.7, 29.6, 29.5, 29.5, 29.5, 26.5, 26.5, 26.1, 25.8, 22.8, 14.3 ppm; MALDI-TOF MS m/z calcd for $C_{85}H_{127}NO_{11}Na$ $[M+Na]^+$ 1360.9, found 1360.9.

^{1}H NMR (400 MHz, $CDCl_3$) spectrum of compound 12

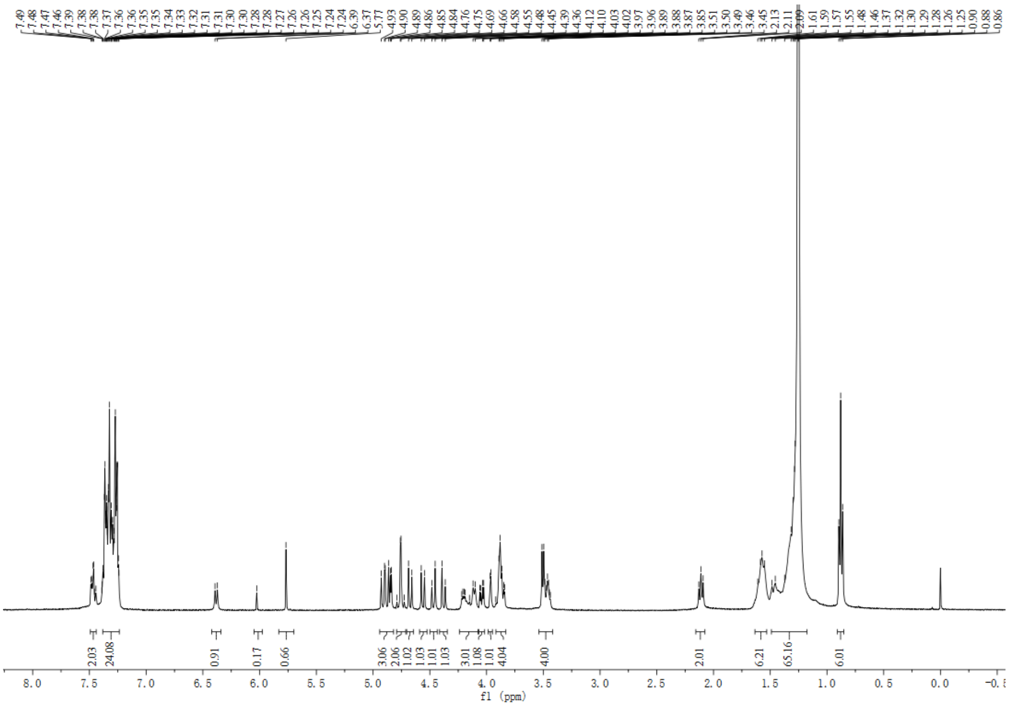

^{13}C NMR (100 MHz, $CDCl_3$) spectrum of compound 12

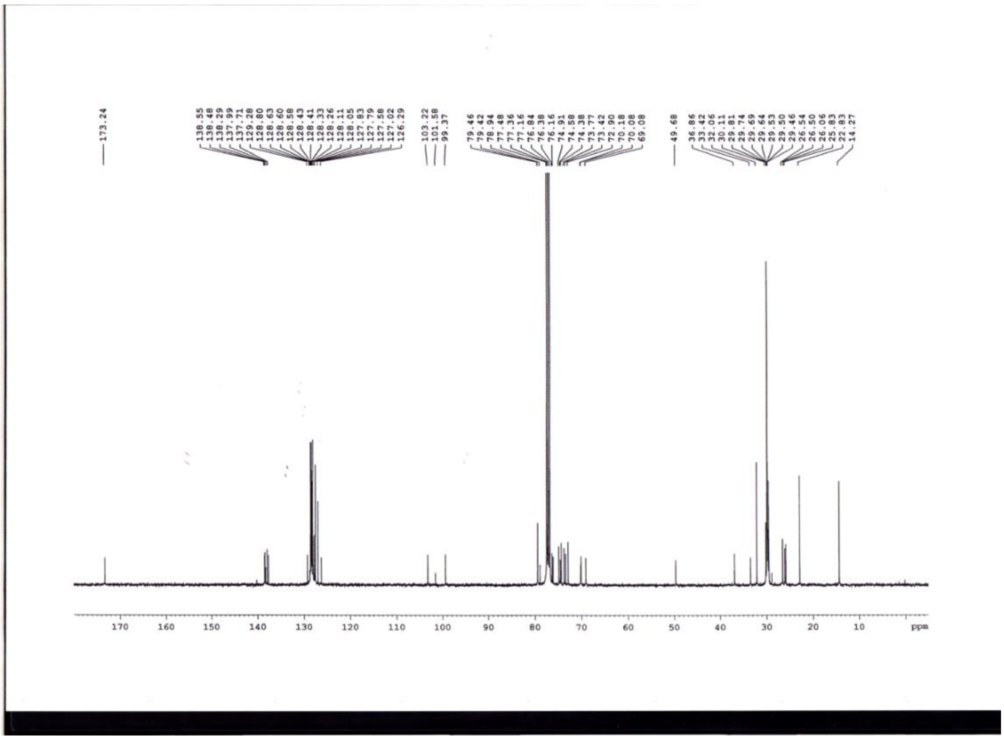

(2S,3S,4R)-1-O-(α-D-galactopyranosyl)-2-N-(12,13-dihydroxyhexacosenoylamino) -1,3,4-octadecanetriol (1)

To a solution of compound **12** (62 mg, 0.046 mmol) in methanol (4 mL) and EtOAc (1 mL) was treated with $Pd(OH)_2/C$ (20 wt% dry basis on carbon, 20 mg) under hydrogen (4 bar) at room temperature for 48 hours. The suspension was filtered through a celite bed and the filter cake was washed several times with $CHCl_3$-CH_3OH(4:1, 4×4 mL). The combined filtrates were concentrated *in vacuo* and purified by silica gel chromatography (20% MeOH in dichloromethane) to yield **1** as a white solid (41 mg, 96%). ^{1}H NMR (400 MHz, Pyridine-d5) δ 8.46 (d, J = 8.6 Hz, 1H), 5.58 (d, J = 3.9 Hz, 1H), 5.28 (d, J = 6.9 Hz, 2H), 4.72 – 4.63 (m, 2H), 4.56 (dd, J = 3.4, 1.2 Hz, 1H), 4.52 (dd, J = 6.6, 5.4 Hz, 1H), 4.46 – 4.37 (m, 4H), 4.32 (d, J = 4.7 Hz, 2H), 4.03 – 3.96 (m, 2H), 2.44 (t, J = 7.5 Hz, 2H), 2.08– 1.98 (m, 1H), 2.02 (m, J = 11.6, 7.6, 4.7 Hz, 2H), 1.90 (d, J = 11.7 Hz, 5H), 1.81 (q, J = 7.6 Hz, 2H), 1.66 (ddd, J = 11.4, 5.9, 2.8 Hz, 3H),

1.50 – 1.16 (m, 57H), 0.91 – 0.84 (m, 6H) ppm; ^{13}C NMR (100 MHz, Pyridine-d5) δ 173.7, 101.9, 77.2, 75.7, 73.4, 72.9, 72.0, 71.4, 70.7, 69.0, 63.1, 51.9, 37.2, 34.8, 34.0, 32.5, 30.8, 30.7, 30.5, 30.4, 30.3, 30.2, 30.1, 30.0, 27.2, 26.9, 26.8, 23.3, 14.7 ppm; HRMS (ESI) m/z calcd for $C_{50}H_{99}NO_{11}Na$ [M+Na]$^+$ 912.7110, found 912.7124.

^{1}H NMR (400 MHz, Pyridine-d5) spectrum of compound 1

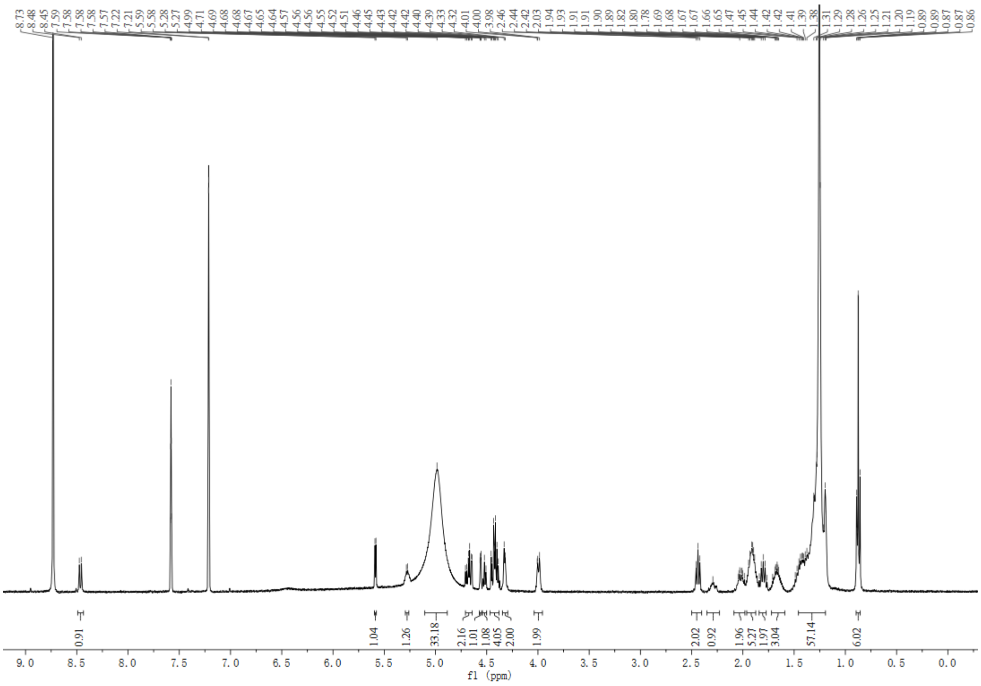

^{13}C NMR (100 MHz, Pyridine-d5) spectrum of compound 1

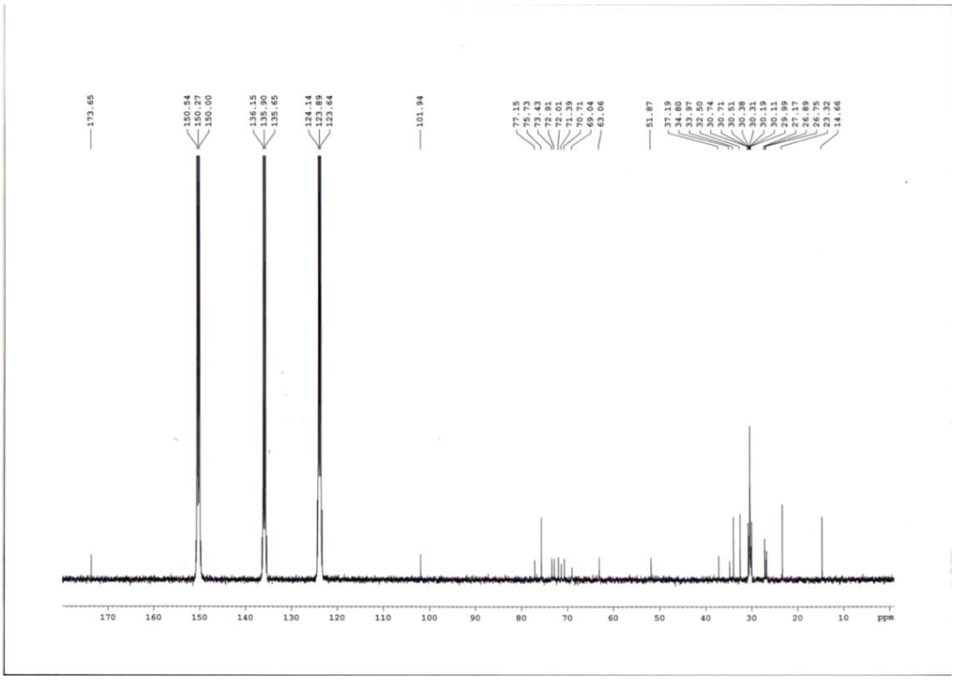

6. Purity analysis of α-GalCer-diol

Analysis conditions:

Pump A: water

Pump B: methanol

Detector: ELSD

Column: Xbridge C-18 4.6 mm x 250 mm, 5 μm

Gradient elution:

**Time（min）**	**Flow（mL/min）**	**A（%）**	**B（%）**
0	1.0	10	90
30	1.0	0	100
40	1.0	0	100
42	1.0	10	90
50	1.0	10	90

Analysis Report

Acquisition: System	Channel name: ELSD signal
Sample name: a-GalCer-diol	Acquisition method: Carbohydrate ELS GM
Sample type: unknown	Processing method: ELSD analysis
Sample size: 50.00 µL	Acquisition time: 2019/12/4 12:17:59 CST
Injection times : 1	Processing time: 2019/12/4 13:21:07 CST

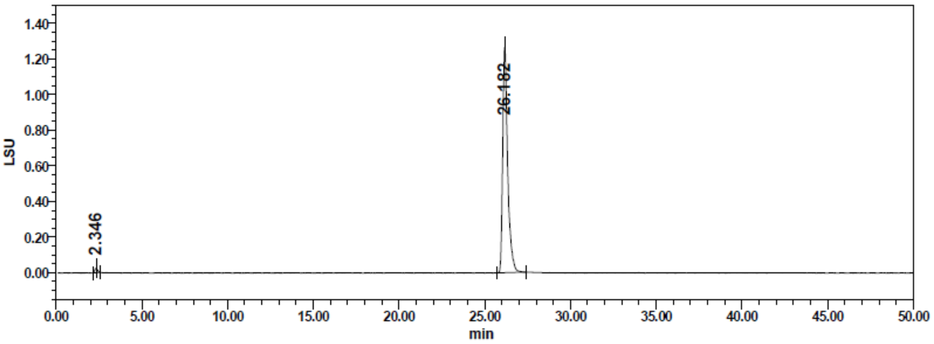

Peak Table

	Retention Time (min)	Peak Area (µV*s)	Peak Area%
1	2.346	298	1.16
2	26.182	25349	98.84

Scheme S7. Purity analysis for leading compound α-GalCer-diol.

7. Supporting information references

[1] T. Tashiro, R. Nakagawa, T. Hirokawa, S. Inoue, H. Watarai, M. Taniguchi, K. Mori, *Tetrahedron Lett.* **2007**, *48*, 3343.

[2] J. Bi, J. Wang, K. Zhou, Y. Wang, M. Fang, Y. Du, *ACS Med. Chem. Lett.* **2015**, *6*, 476.

[3] M. Trappeniers, K. Van Beneden, T. Decruy, U. Hillaert, B. Linclau, D. Elewaut, S. Van Calenbergh, *J. Am. Chem. Soc.* **2008**, *130*, 16468.

[4] W. Zhang, C. Xia, J. Nadas, W. Chen, L. Gu, P.G. Wang, *Bioorg. Med. Chem.* **2011**, *19*, 2767.

[5] J. Janssens, T. Decruy, K. Venken, T. Seki, S. Krols, J. Van der Eycken, M. Tsuji, D. Elewaut, S. Van Calenbergh, *ACS Med. Chem. Lett.* **2017**, *8*, 642.

[6] J. Janssens, A. Bitra, J. Wang, T. Decruy, K. Venken, J. van der Eycken, D. Elewaut, D.M. Zajonc, S. van Calenbergh, *ChemMedChem* **2019**, *14*, 147.

[7] J. Schmieg, G. Yang, R.W. Franck, M. Tsuji, *J. Exp. Med.* **2003**, *198*, 1631.

[8] R.W. Franck, M. Tsuji, *Acc. Chem. Res.* **2006**, *39*, 692.

[9] M. Fujio, D. Wu, R. Garcia-Navarro, D.D. Ho, M. Tsuji, C.H. Wong, *J. Am. Chem. Soc.* **2006**, *128*, 9022.

[10] Y.J. Chang, J.R. Huang, Y.C. Tsai, J.T. Hung, D. Wu, M. Fujio, C.H. Wong, A.L. Yu, *Proc. Natl. Acad. Sci. U. S. A.* **2007**, *104*, 10299.

[11] M.I. Hossain, S. Hanashima, T. Nomura, S. Lethu, H. Tsuchikawa, M. Murata, H. Kusaka, S. Kita, K. Maenaka, *Bioorg. Med. Chem.* **2016**, *24*, 3687.

[12] D.G. Pellicci, O. Patel, L. Kjer-Nielsen, S.S. Pang, L.C. Sullivan, K. Kyparissoudis, A.G. Brooks, H.H. Reid, S. Gras, I.S. Lucet, R. Koh, M.J. Smyth, T. Mallevaey, J.L. Matsuda, L. Gapin, J. McCluskey, D.I. Godfrey, J. Rossjohn, *Immunity* **2009**, *31*, 47.
